# Correction: Ncongwane et al. Labdane Diterpenoids from *Leonotis ocymifolia* with Selective Cytotoxic Activity Against HCC70 Breast Cancer Cell Line. *Diseases* 2025, *13*, 140

**DOI:** 10.3390/diseases14060197

**Published:** 2026-06-02

**Authors:** Jane Busisiwe Ncongwane, Comfort Nkambule, Gerda Fouche, Douglas Kemboi, Nyeleti Vukea, Candace Davison, Jo-Anne de la Mare, Vuyelwa Jacqueline Tembu

**Affiliations:** 1 Department of Chemistry, Tshwane University of Technology, Private Bag X680, Pretoria 0001, South Africa; ncongwanejb@tut.ac.za (J.B.N.); nkambulecm@tut.ac.za (C.N.); 2 Department of Chemistry, University of Pretoria, Pretoria 0001, South Africa; gerda.fouche@up.ac.za; 3 School of Science and Technology, University of Kabianga, Kericho 2030-20200, Kenya; 4 Department of Biochemistry and Microbiology, Rhodes University, Grahamstown 6140, South Africa; nyeletivukea@gmail.com (N.V.); candacedavison9@gmail.com (C.D.); j.delamare@ru.ac.za (J.-A.d.l.M.)

## Addition of an Author

Candace Davison was not included as an author in the original publication [1]. The corrected Author Contributions Statement appears here. Author Contributions: Conceptualization, J.B.N., V.J.T., G.F. and C.N.; methodology J.B.N., V.J.T., G.F., N.V. and C.D.; writing—original draft preparation, J.B.N.; writing—review and editing, J.B.N., V.J.T., G.F., J.-A.d.l.M., C.N. and D.K.; supervision, V.J.T., G.F., J.-A.d.l.M. and C.N.; funding acquisition, V.J.T., G.F., J.-A.d.l.M. and C.N. All authors have read and agreed to the published version of the manuscript.

## Correction of Authorship Sequence

In the published publication [1], there was an error regarding the authorship sequence. The correct authorship sequence appears below and affiliation re-ordered.

Jane Busisiwe Ncongwane ^1^, Comfort Nkambule ^1^, Gerda Fouche ^2^, Douglas Kemboi ^1,3,^*, Nyeleti Vukea ^4^, Candace Davison ^4^, Jo-Anne de la Mare ^4^ and Vuyelwa Jacqueline Tembu ^1,^*

## Text Correction

There was an error in the original publication [1]. The method stated was incorrect.

A correction has been made to Materials and Methods, Cytotoxic Assay, the first Paragraph:

The cytotoxicity and IC_50_ values of the isolated compounds were evaluated in three cell lines, namely, the HCC70 basal triple-negative breast cancer (TNBC) cell line (ATCC: CRL-2315), the hormone receptor-positive breast cancer MCF-7 cell line (ATCC: HTB-22) and the non-tumorigenic breast epithelial cell line MCF12A (ATCC: CRL-10782) using the resazurin assay. The cells were seeded at a density of 5000 cells/well in a 96-well plate and allowed to attach overnight at 37 °C and 9% CO_2_. The cells were then treated with DMSO as vehicle control (0.2% vM), paclitaxel (Sigma-Aldrich, St. Louis, USA) as a positive control (0.32 to 200 nm) and a six-point range concentration (0.32 to 200 μM) of the isolated pure compounds and incubated for 96 h at 37 DC in a humidified 9% CO_2_ incubator. Resazurin (0.54 nM) was added to the cells, and the cells were incubated for 2–4 h at 37 °C in 9% CO_2_. Fluorescence readings were obtained on a Spectramax spectrophotometer (excitation and emission wavelength set to 560 nm and 590 nm, respectively) [23]. The experiment was performed in triplicate, and the data was analyzed using GraphPad Prism Inc., (Version 4, USA) with a half-maximal inhibitory concentration (IC_50_ values) determined by non-linear regression. A selectivity index (SI) was determined for each compound to determine its therapeutic significance in cancerous cells. It was calculated as follows:

$$Selectivity\;index\;(SI)\;=\;\frac{IC50\;of\;compound\;in\;MCF12A}{IC50\;of\;compound\;in\;cancer\;line}$$

## Reference Correction

In the original publication, there was an error in the Reference list. The reference [23] should be replaced with: “Mbaba, M.; Dingle, L.M.K.; Cash, D.; de la Mare, J.; Laming, D.; Taylor, D.; Hoppe, H.C.; Edkins, A.L.; Khanye, S.D. Repurposing a polymer precursor: Synthesis and in vitro medicinal potential of ferrocenyl 1,3-benzoxazine derivatives. *Eur. J. Med. Chem.* **2020**, *187*, 111924.’’.

The authors state that the scientific conclusions are unaffected. This correction was approved by the Academic Editor. The original publication has also been updated.
