# Supplementary material for: Labdane Diterpenoids from Leonotis ocymifolia with Selective Cytotoxic Activity Against HCC70 Breast Cancer Cell Line
Source: Diseases. 2025 May 1;13(5):140. doi: 10.3390/diseases13050140 (PMC12110735; doi:10.3390/diseases13050140)
Supplement: Supplementary file 1 [file diseases-13-00140-s001.zip › diseases-3476534-supplementary.pdf]

Labdane diterpenoids from Leonotis ocymifolia with selective cytotoxic activity against HCC70 breast cancer cell line

Jane Busisiwe Ncongwane 1*, Vuyelwa Jacqueline Tembu1, Comfort Nkambule 1, Douglas Kemboi 1, 4, Gerda Fouche 2, Nyeleti Vukea 3 and Jo-Anne de la Mare 3

1 Department of Chemistry, Tshwane University of Technology, Private Bag X680 Pretoria, 0001, South Africa ; ncongwanejb@tut.ac.za (J.B.N); TembuVJ@tut.ac.za (V.J.T.); nkambulecm@tut.ac.za (C.N); kemboidouglas01@gmail.com (D.K.M.)

2 Department of Chemistry, University of Pretoria, Pretoria, 0001, South Africa; gerda.fouche@up.ac.za

3 Department of Biochemistry and Microbiology, Biomedical Biotechnology Research Unit, Rhodes University, Grahamstown, 6140, South Africa

4 School of Science and Technology. University of Kabianga, 2030-20200, Kericho, Kenya

* Correspondence: ncongwanejb@tut.ac.za; TembuVJ@tut.ac.za (V.J.T.); Tel.: +27-12-382-6288 (V.J.T.)

Abstract

Leonotis ocymifolia is a shrub widely used in traditional medicine to alleviate illnesses such as wounds, abdominal pain, headache, hypertension, malaria, asthma, diabetes, and eczema. Compounds from *Leonotis ocymifolia* were characterised using spectroscopic data such as IR, 1D and 2D NMR and MS spectrometry and evaluated for cytotoxic activity against triple negative breast cancer (HCC70), hormone receptor-positive breast cancer (MCF-7), and non-tumorigenic mammary epithelial cell lines (MCF-12A). A novel bis-spirolabdane, 13S-nepetaefolin (**1**), together with five known labdane diterpenoids, nepetaefolin (**2**), dubiin (**3**), nepetaefuran (**4**), leonotin (**5**) and leonotinin (**6**) from the genus Leonotis were isolated. Overall, the labdane diterpenoids showed selective activity toward triple negative breast cancer cells (HCC70). Of the compounds extracted, 13S-nepetaefolin demonstrated the greatest cytotoxic activity with an IC_{50} of 24.65 μM against HCC70 cells, however, it was equally cytotoxic to non-tumorigenic MCF-12A breast cells (IC_{50} of 26.55 μM) whereas its isomer showed no activity. This suggests that stereochemistry might have an effect on the cytotoxic activity of the bis-spirolabdane diterpenoids.

Keyword

Leonotis ocymifolia, labdane diterpenoids, MCF-7, HCC70, cytotoxic activity

Contents

Appendix 1: ^{1}H NMR spectrum of 13*S*-nepetaefolin (1) in $CDCl_3$ 3
Appendix 2: ^{13}C NMR spectrum of 13S nepetaefolin (1) in $CDCl_3$ 4
Appendix 3: HSQC spectrum of 13S nepetaefolin (1) in $CDCl_3$ 5
Appendix 4: HMBC spectrum of 13S nepetaefolin (1) in $CDCl_3$ 6
Appendix 5: NOESY spectrum of 13S nepetaefolin (1) in $CDCl_3$ 7
Appendix 6: IR spectrum of 13S nepetaefolin (1) 8
Appendix 7: MS spectrum of 13S nepetaefolin (1) 9
Appendix 8: ^{1}H NMR spectrum of nepetaefolin (2) in $CDCl_3$ 10
Appendix 9: ^{13}C NMR spectrum of nepetaefolin (2) in $CDCl_3$ 11
Appendix 10: IR spectrum of nepetaefolin (2) 12
Appendix 11: MS spectrum of nepetaefolin (2) 13
Appendix 12: ^{1}H NMR spectrum of dubiin (3) in $CDCl_3$ 14
Appendix 13: ^{13}C NMR spectrum of dubiin (3) in $CDCl_3$ 15
Appendix 14: IR spectrum of dubiin (3) 16
Appendix 15: MS spectrum of dubiin (3) 17
Appendix 16: ^{1}H NMR spectrum of nepetaefuran (4) in $CDCl_3$ 18
Appendix 17: ^{13}C NMR spectrum of nepetaefuran (4) in $CDCl_3$ 19
Appendix 18: IR spectrum of nepetaefuran (4) 20
Appendix 19: MS spectrum of nepetaefuran (4) 21
Appendix 20: ^{1}H NMR spectrum of leonotin (5) in $CDCl_3$ 22
Appendix 21: ^{13}C NMR spectrum of leonotin (5) in $CDCl_3$ 23
Appendix 22: IR spectrum of leonotin (5) 24
Appendix 23: MS spectrum of leonotin (5) 25
Appendix 24: ^{1}H NMR spectrum of leonotinin (6) in $CDCl_3$ 26
Appendix 25: ^{13}C NMR spectrum of leonotinin (6) in $CDCl_3$ 27
Appendix 26: IR spectrum of leonotinin (6) 28
Appendix 27: MS spectrum of leonotinin (6) 29

Appendix 1: ^{1}H NMR spectrum of 13*S*-nepetaefolin (1) in $CDCl_3$

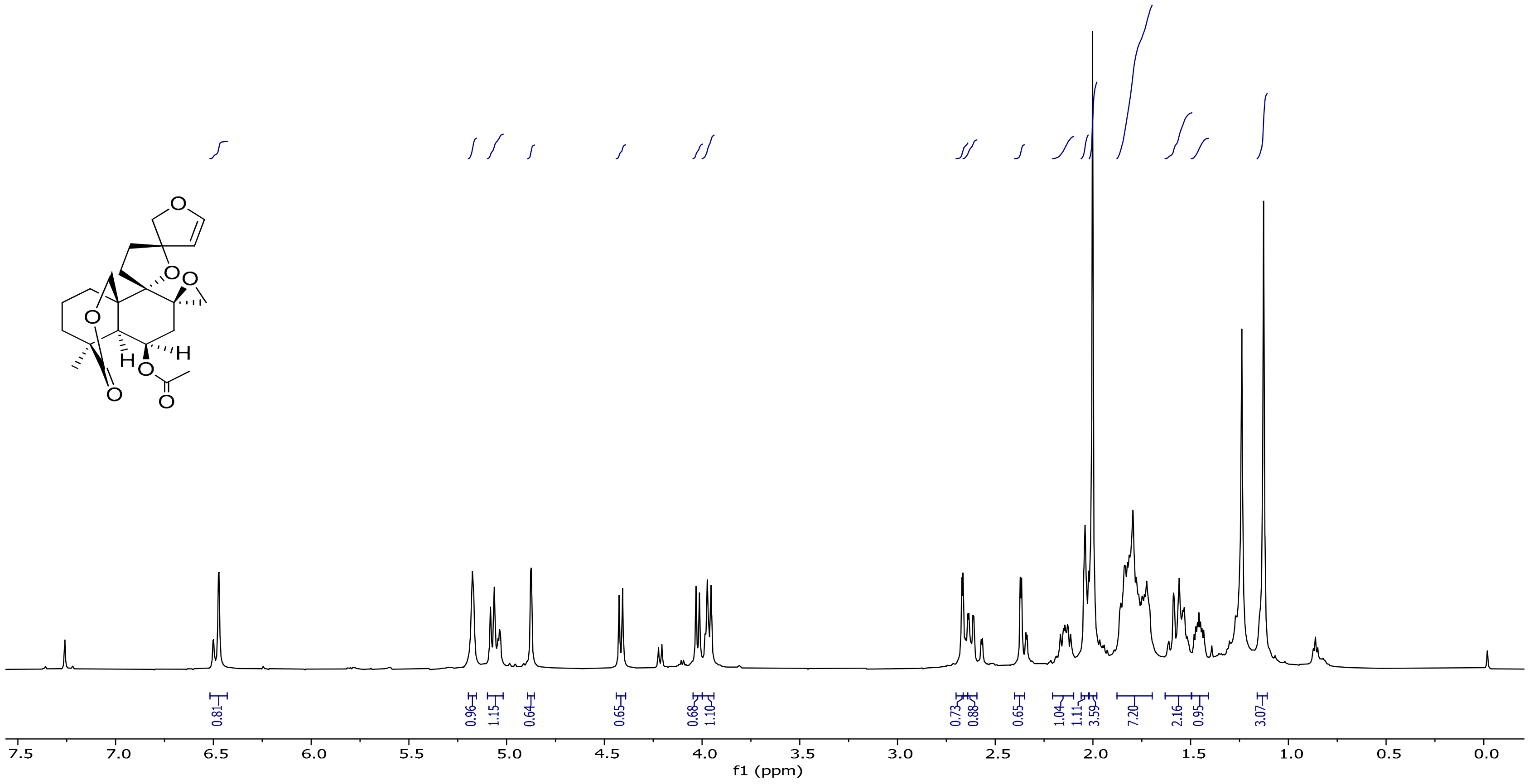

Spectrum A1: ^{1}H NMR spectrum of compound 1 (13*S*-Nepetaefolin)

Appendix 2: ^{13}C NMR spectrum of 13S nepetaefolin (1) in $CDCl_3$

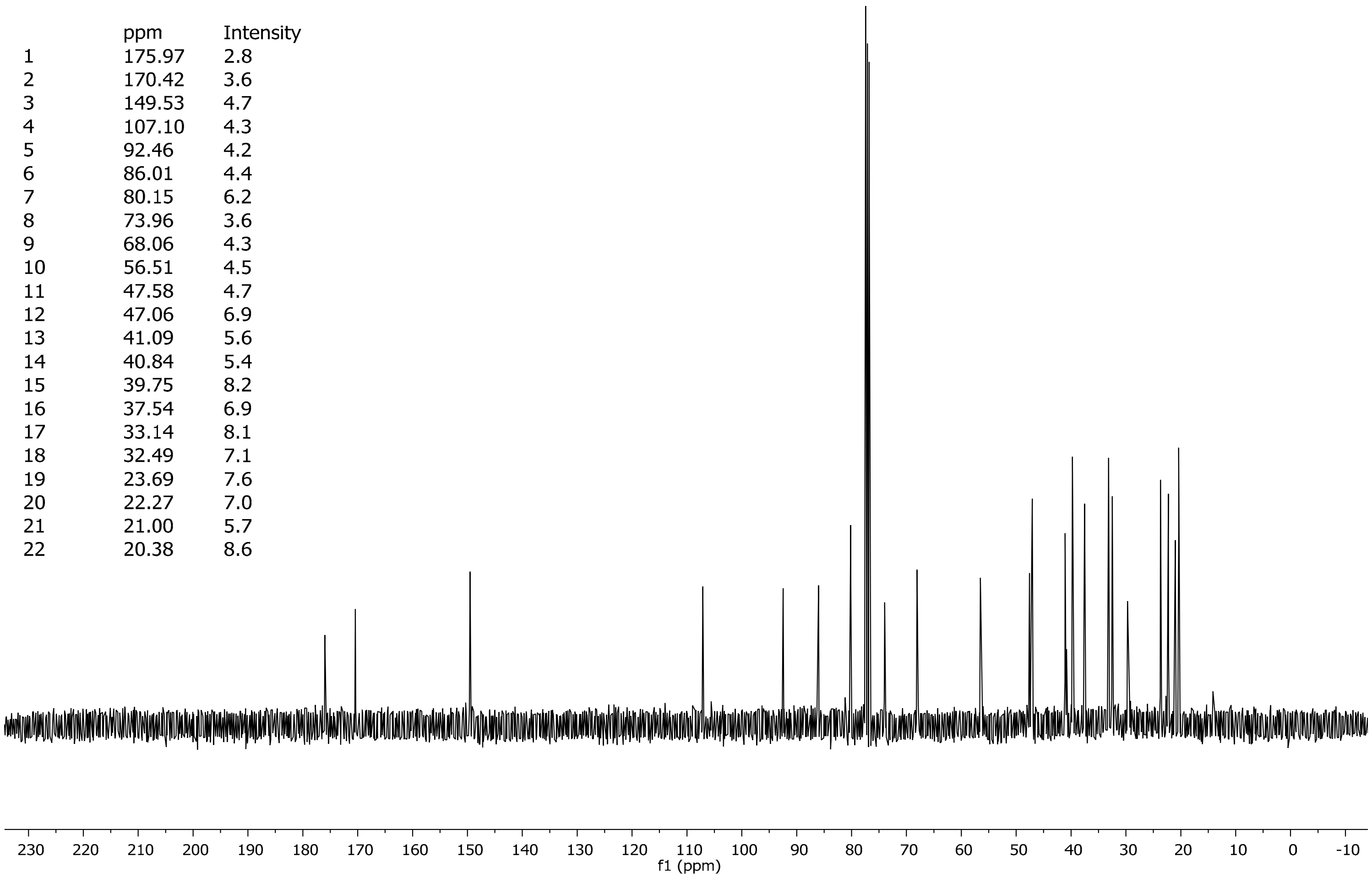

Spectrum A2: ^{13}C NMR spectrum of compound 1 (13*S*-Nepetaefolin)

Appendix 3: HSQC spectrum of 13S nepetaefolin (1) in $CDCl_3$

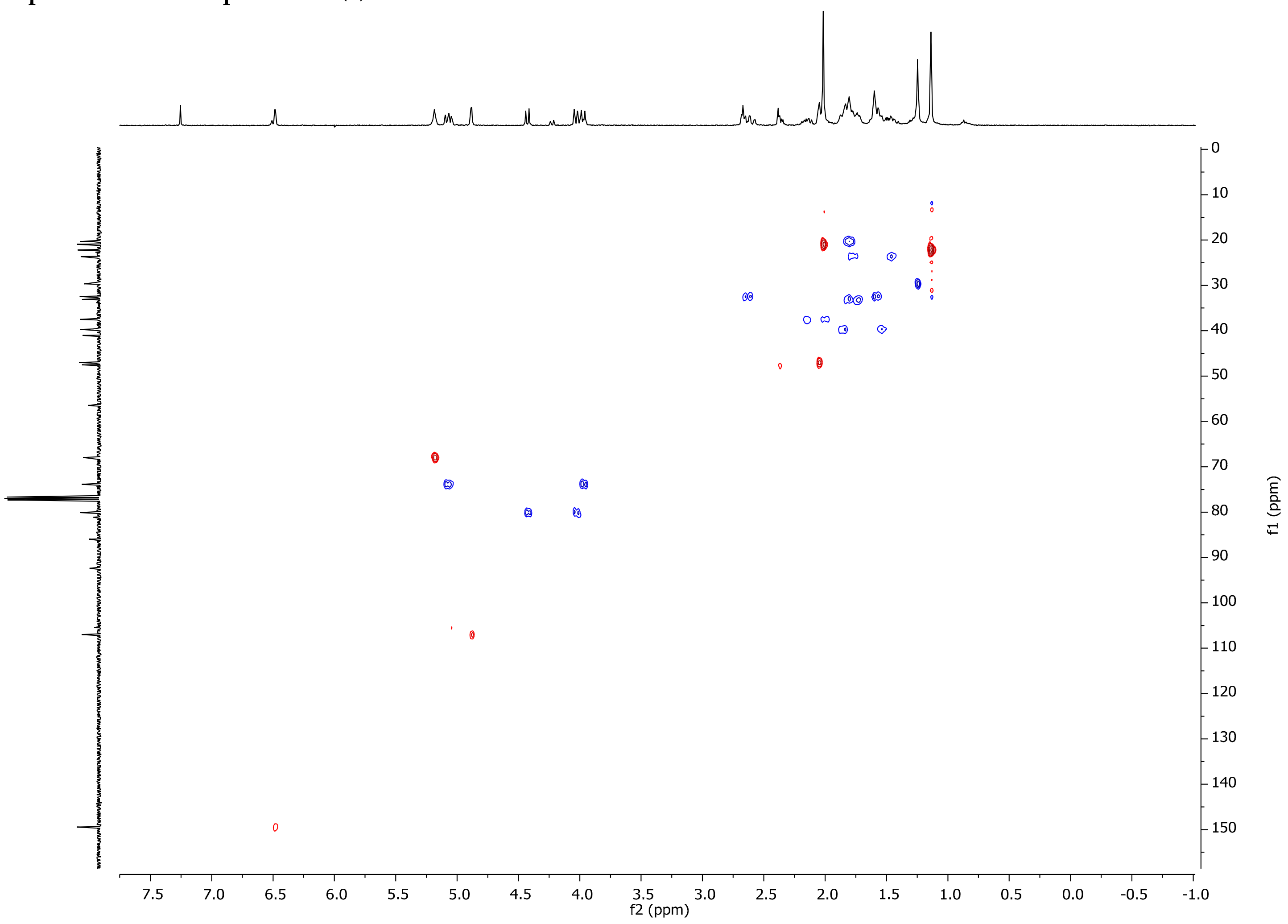

Spectrum A3: HSQC spectrum of compound 1 (13*S*-Nepetaefolin)

Appendix 4: HMBC spectrum of 13S nepetaefolin (1) in $CDCl_3$

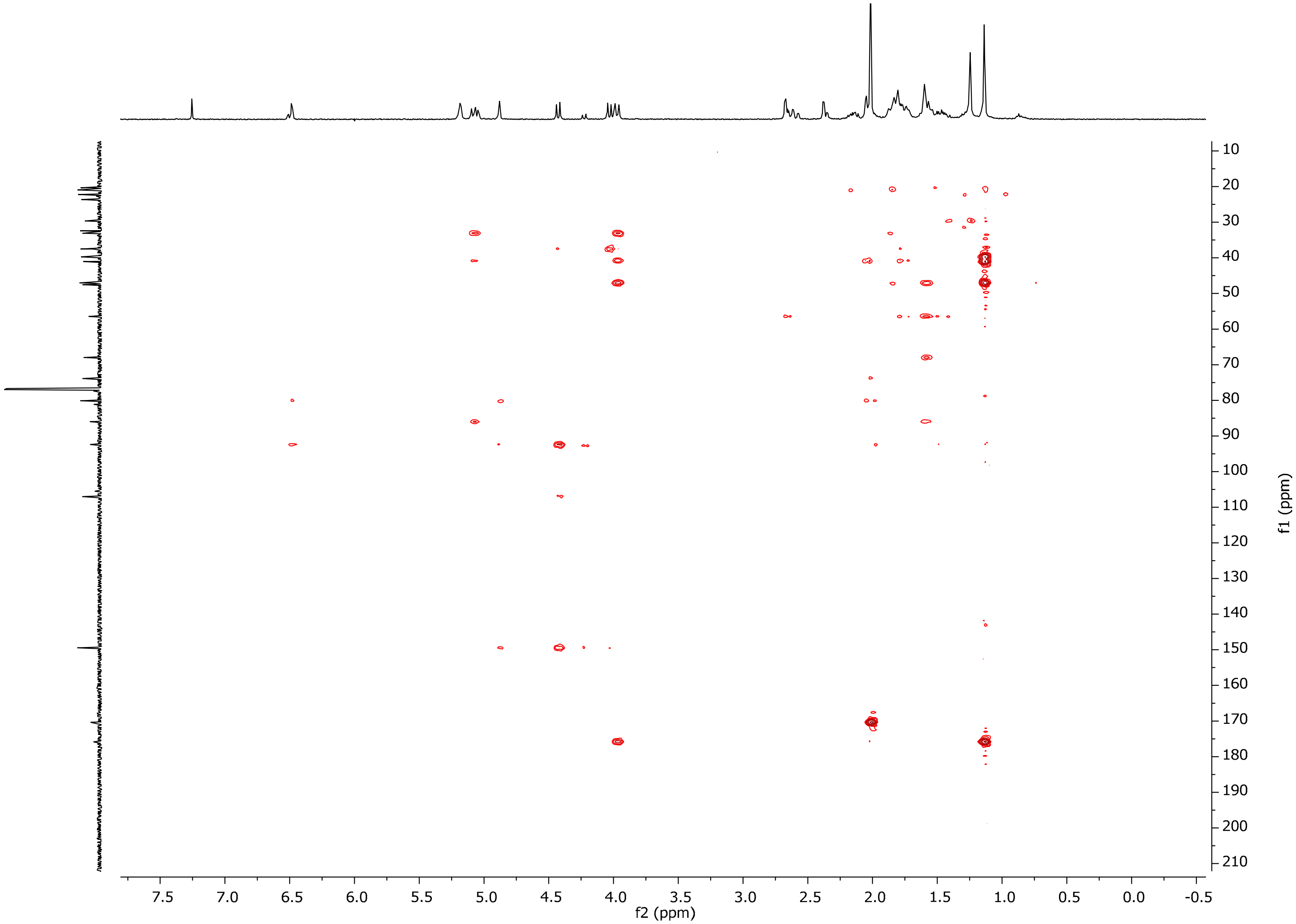

Spectrum A4: HMBC NMR spectrum of compound 1 (13*S*-Nepetaefolin)

Appendix 5: NOESY spectrum of 13S nepetaefolin (1) in $CDCl_3$

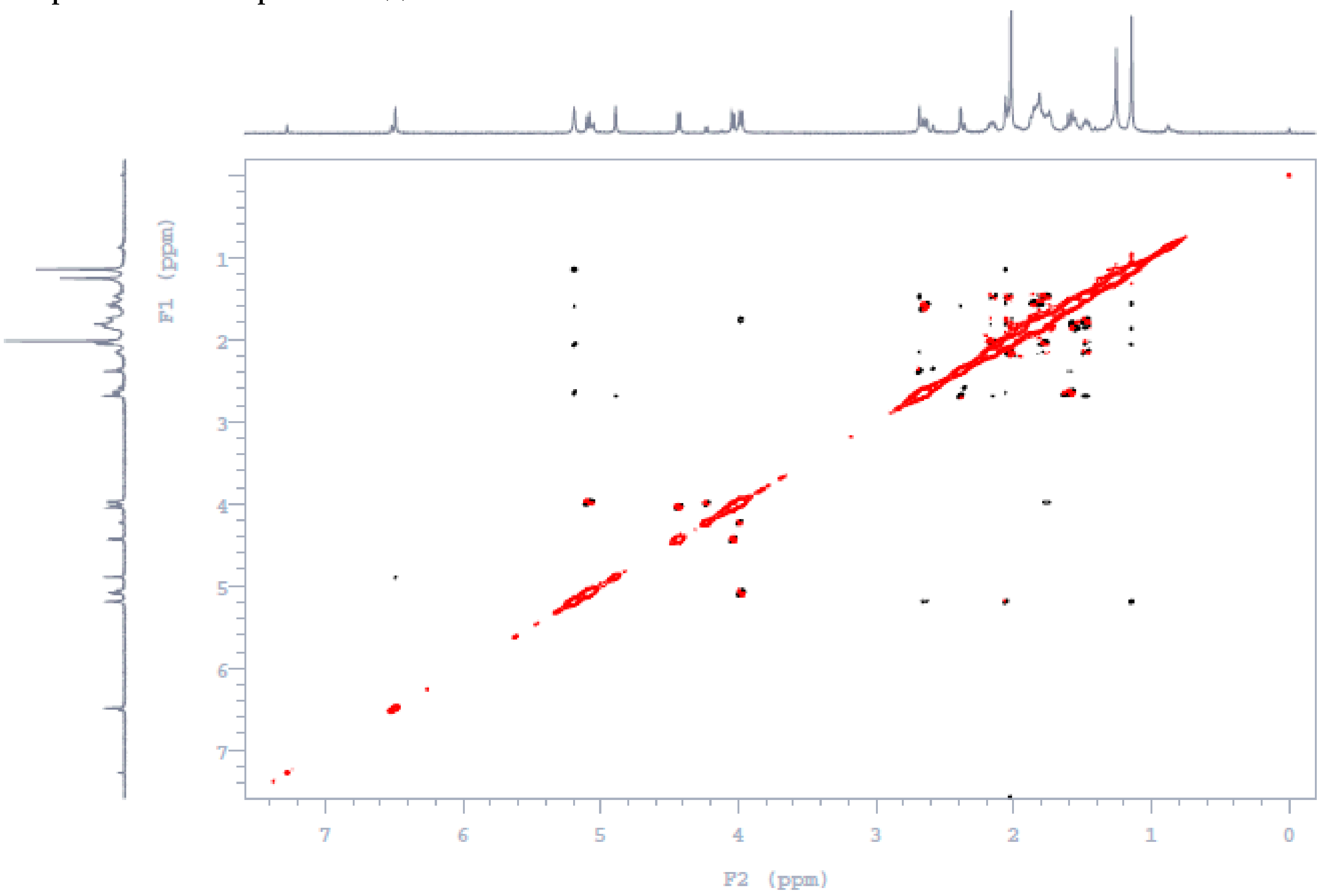

Spectrum A6: NOESY NMR spectrum of compound 1 (13*S*-Nepetaefolin)

Appendix 6: IR spectrum of 13S nepetaefolin (1)

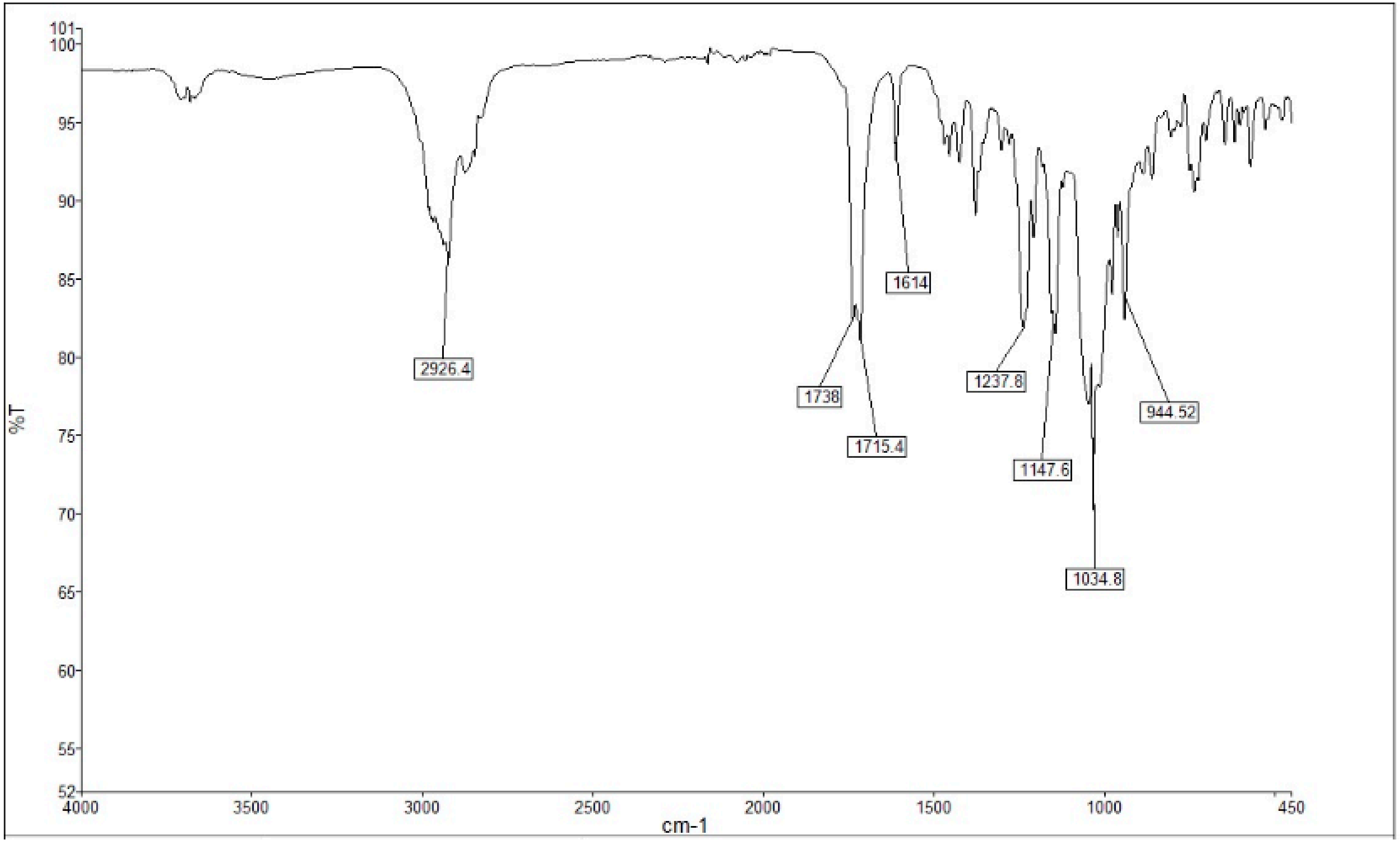

Spectrum A7: IR spectrum of compound 1 (13*S*-Nepetaefolin)

Appendix 7: MS spectrum of 13S nepetaefolin (1)

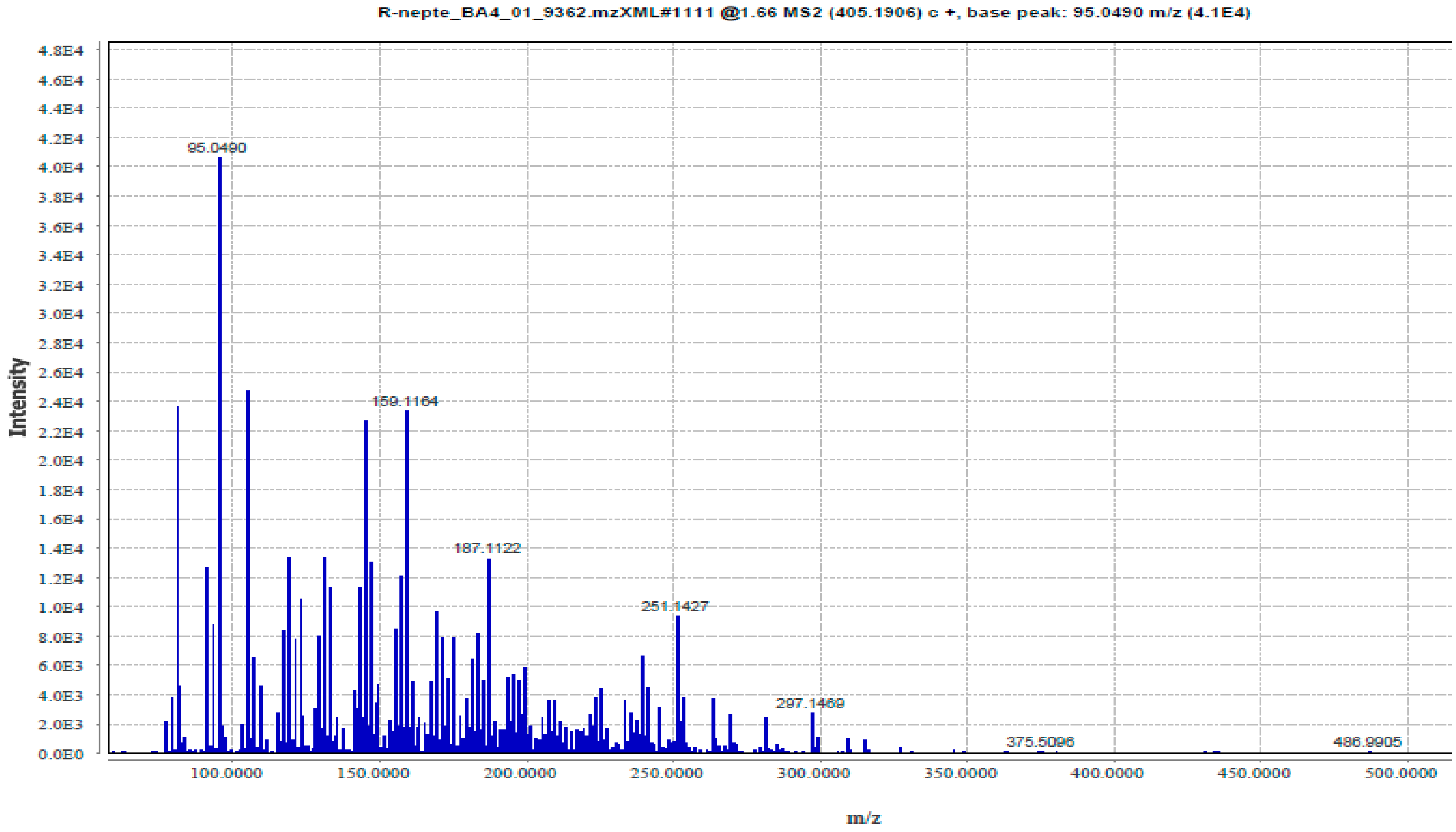

Spectrum A8: MS spectrum of compound 1 (13*S*-Nepetaefolin)

Appendix 8: ^{1}H NMR spectrum of nepetaefolin (2) in $CDCl_3$

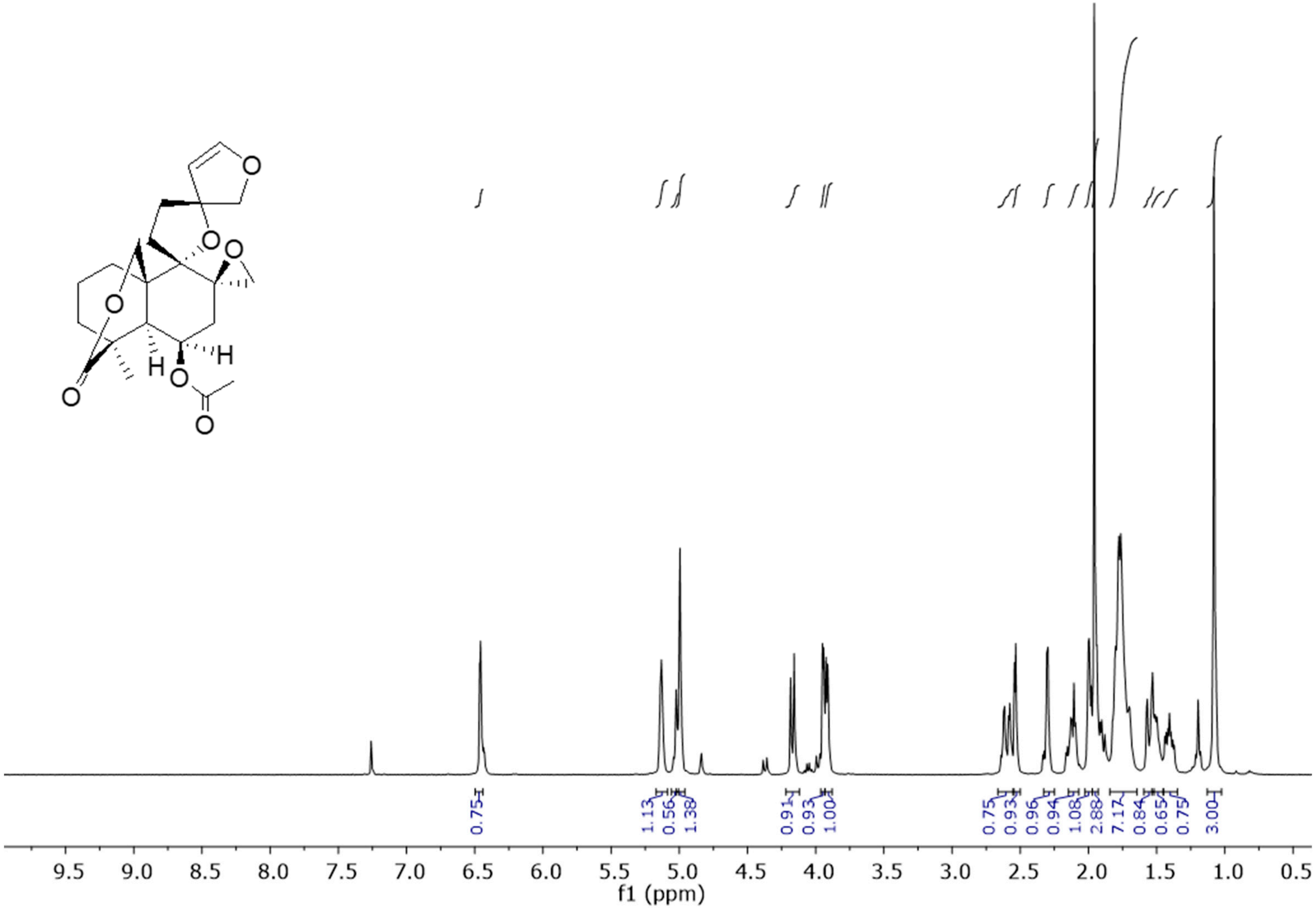

Spectrum B1: ^{1}H NMR spectrum of compound 2 (nepetaefolin)

Appendix 9: ^{13}C NMR spectrum of nepetaefolin (2) in $CDCl_3$

	ppm	Intensity
1	175.79	37.8
2	170.26	47.1
3	149.37	35.3
4	105.44	28.4
5	92.71	59.2
6	85.95	55.3
7	81.06	41.7
8	73.77	45.7
9	67.87	32.1
10	56.07	62.3
11	47.12	41.9
12	46.89	85.2
13	40.99	84.5
14	40.71	66.7
15	39.59	76.0
16	37.46	80.1
17	33.24	80.5
18	32.45	69.7
19	23.50	77.7
20	22.17	73.5
21	20.90	78.3
22	20.31	81.6

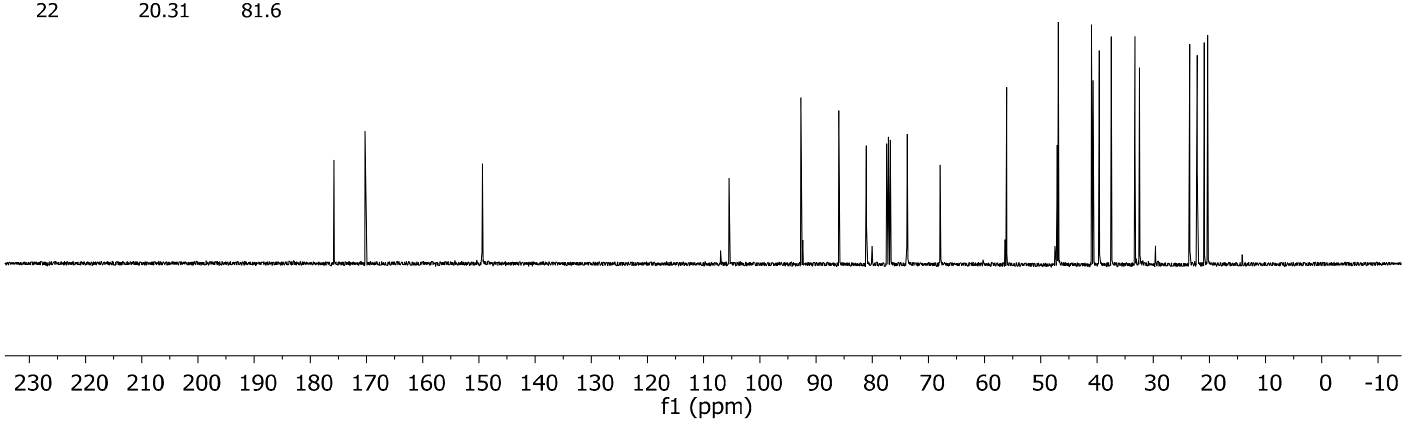

Spectrum B2: ^{13}C NMR spectrum of compound 2 (nepetaefolin)

Appendix 10: IR spectrum of nepetaefolin (2)

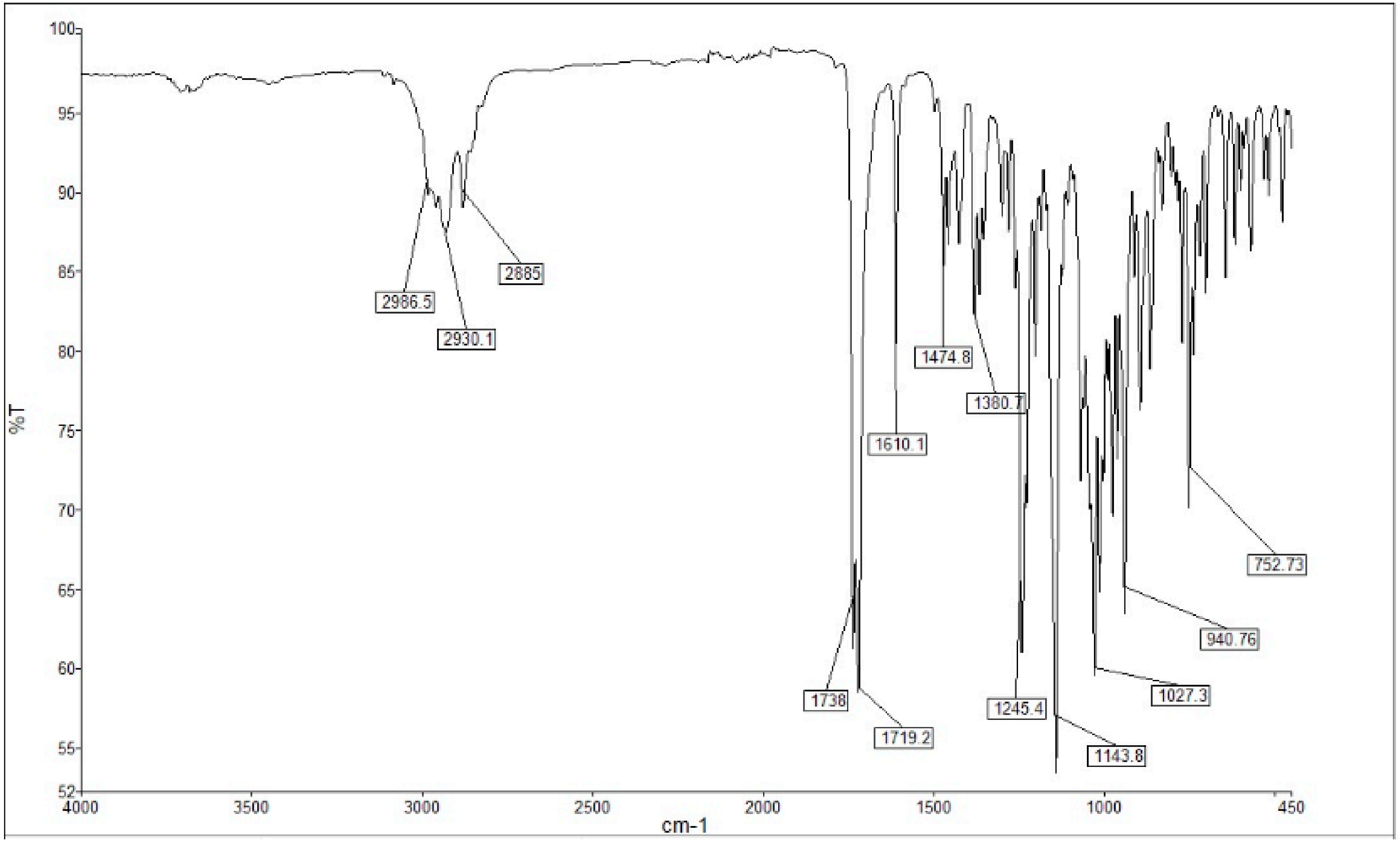

Spectrum B3: IR spectrum of compound 2 (nepetaefolin)

Appendix 11: MS spectrum of nepetaefolin (2)

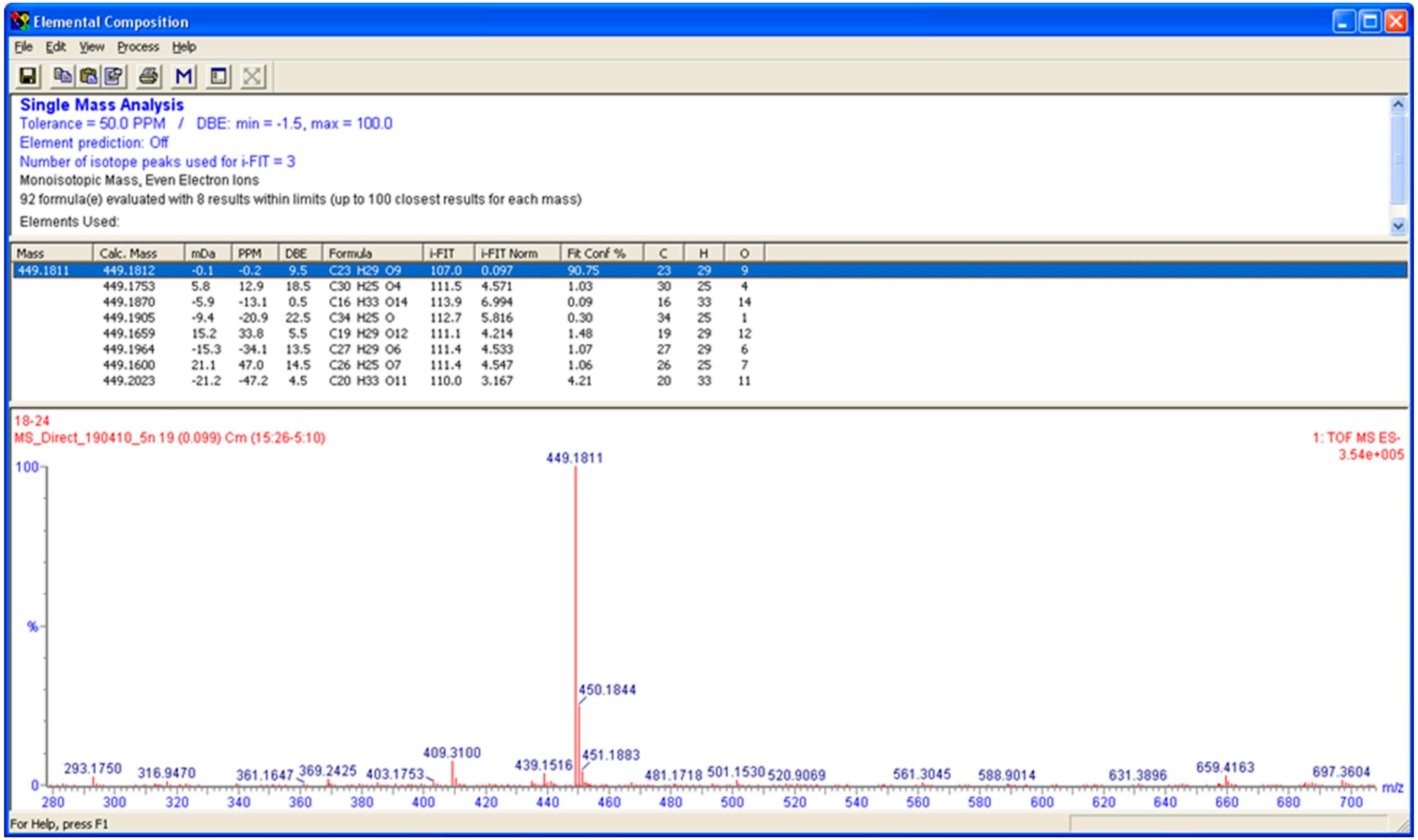

Mass	Calc. Mass	mDa	PPM	DBE	Formula	i-FIT	i-FIT Norm	Fit Conf %	C	H	O
449.1811	449.1812	-0.1	-0.2	9.5	C23 H29 O9	107.0	0.097	90.75	23	29	9
	449.1753	5.8	12.9	18.5	C30 H25 O4	111.5	4.571	1.03	30	25	4
	449.1870	-5.9	-13.1	0.5	C16 H33 O14	113.9	6.994	0.09	16	33	14
	449.1905	-9.4	-20.9	22.5	C34 H25 O	112.7	5.816	0.30	34	25	1
	449.1659	15.2	33.8	5.5	C19 H29 O12	111.1	4.214	1.48	19	29	12
	449.1964	-15.3	-34.1	13.5	C27 H29 O6	111.4	4.533	1.07	27	29	6
	449.1600	21.1	47.0	14.5	C26 H25 O7	111.4	4.547	1.06	26	25	7
	449.2023	-21.2	-47.2	4.5	C20 H33 O11	110.0	3.167	4.21	20	33	11

Spectrum B4: TOF-MS spectrum of compound 2 (nepetaefolin)

Appendix 12: ^{1}H NMR spectrum of dubiin (3) in $CDCl_3$

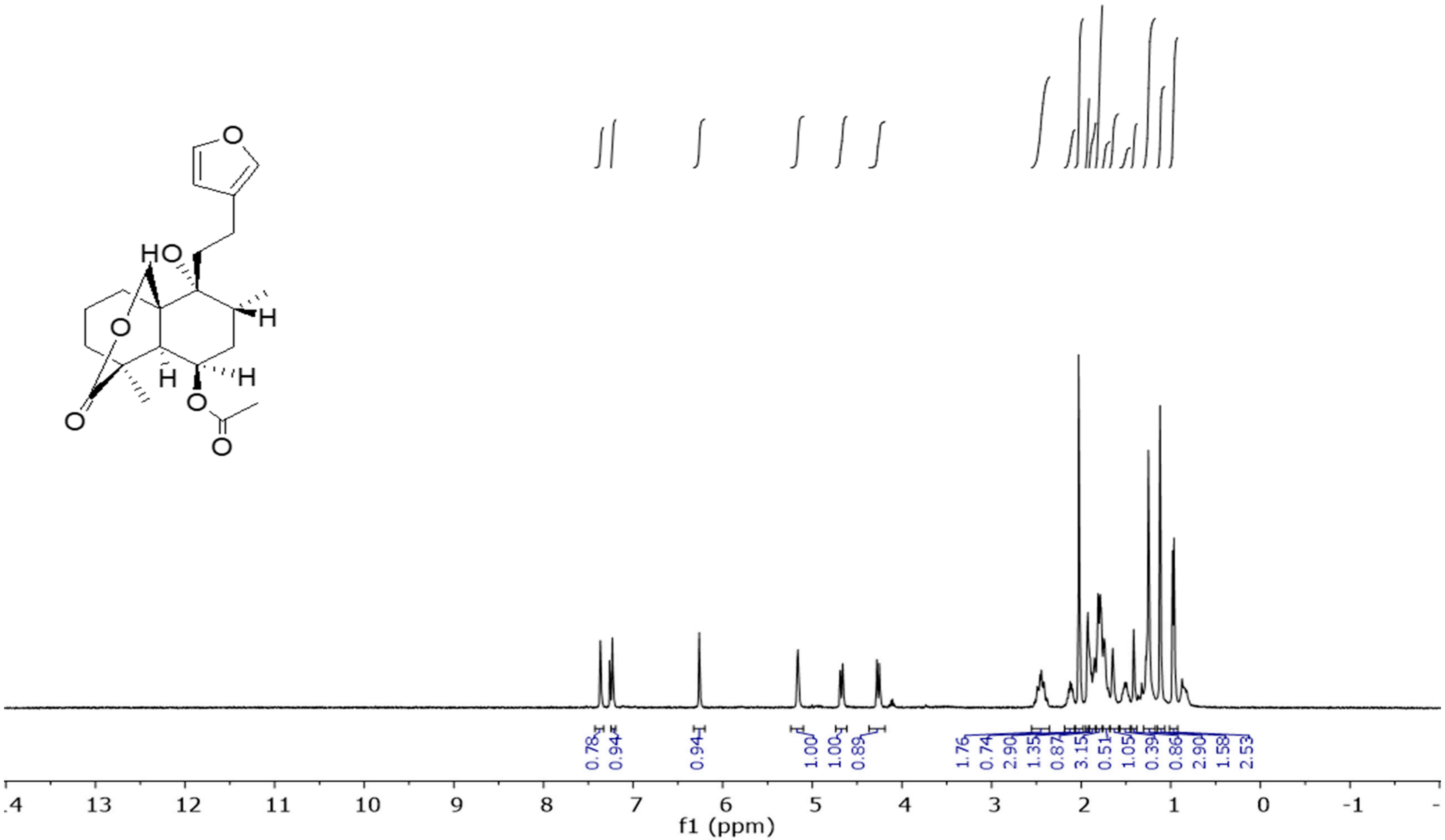

Spectrum C1: ^{1}H NMR spectrum of compound 3 (Dubiin)

Appendix 13: ^{13}C NMR spectrum of dubiin (3) in $CDCl_3$

	ppm	Intensity
1	170.20	3.6
2	143.24	7.5
3	138.55	7.7
4	124.39	3.6
5	110.50	6.8
6	75.83	6.5
7	74.91	5.4
8	68.33	6.5
9	46.80	7.7
10	41.09	5.1
11	40.97	4.9
12	39.72	6.5
13	35.05	6.7
14	33.33	7.1
15	32.87	6.8
16	30.22	7.2
17	29.67	4.4
18	22.37	6.7
19	21.01	7.1
20	20.51	7.6
21	20.39	6.5
22	15.61	7.2

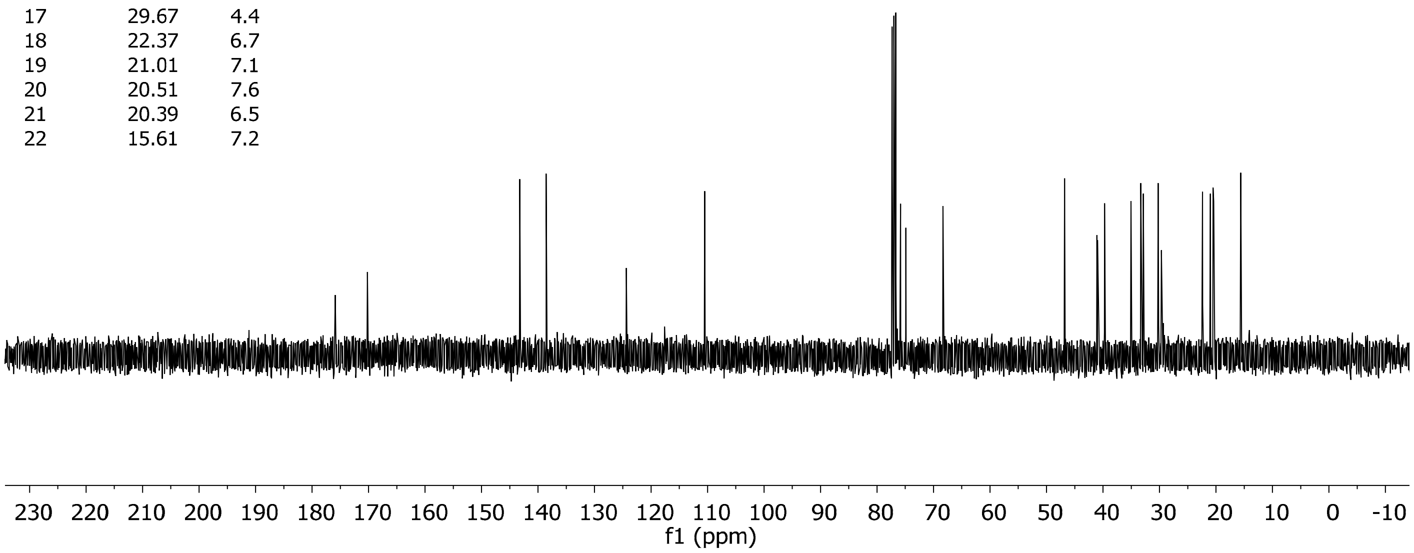

Spectrum C2: ^{13}C NMR spectrum of compound 3 (Dubiin)

Appendix 14: IR spectrum of dubiin (3)

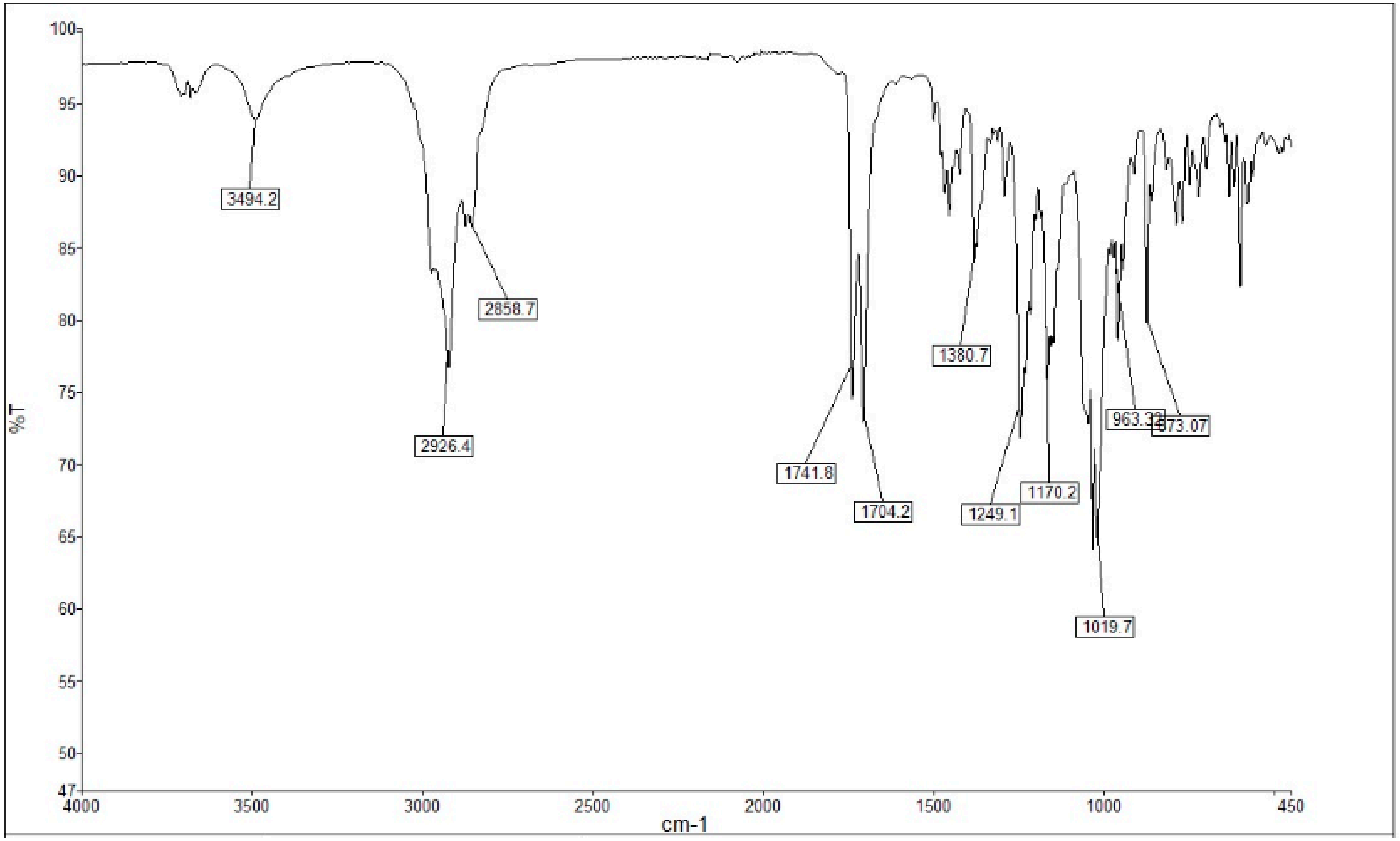

Spectrum C3: IR spectrum of compound 3 (Dubiin)

Appendix 15: MS spectrum of dubiin (3)

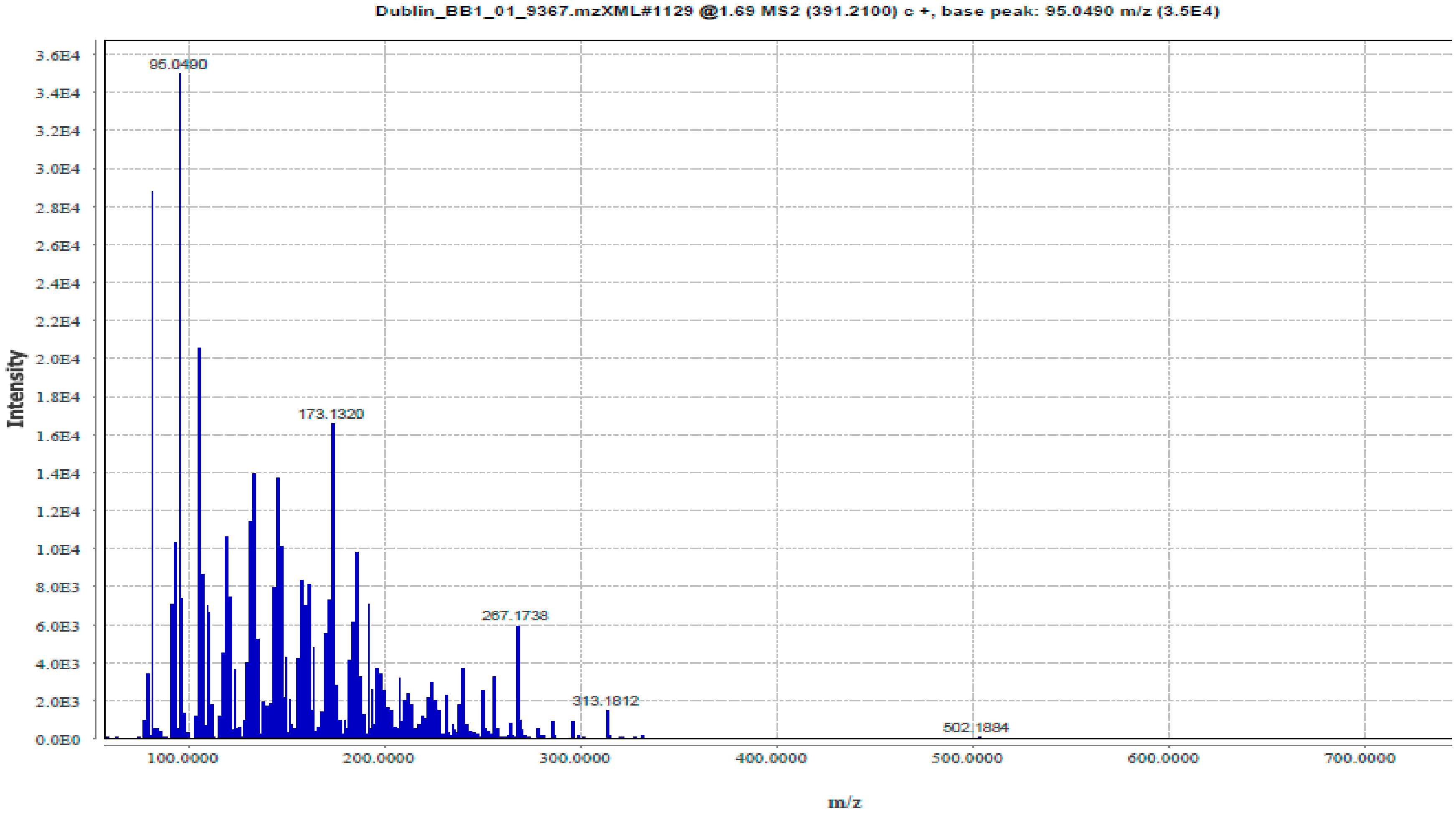

Spectrum C4: MS spectrum of compound 3 (Dubiin)

Appendix 16: ^{1}H NMR spectrum of nepetaefuran (4) in $CDCl_3$

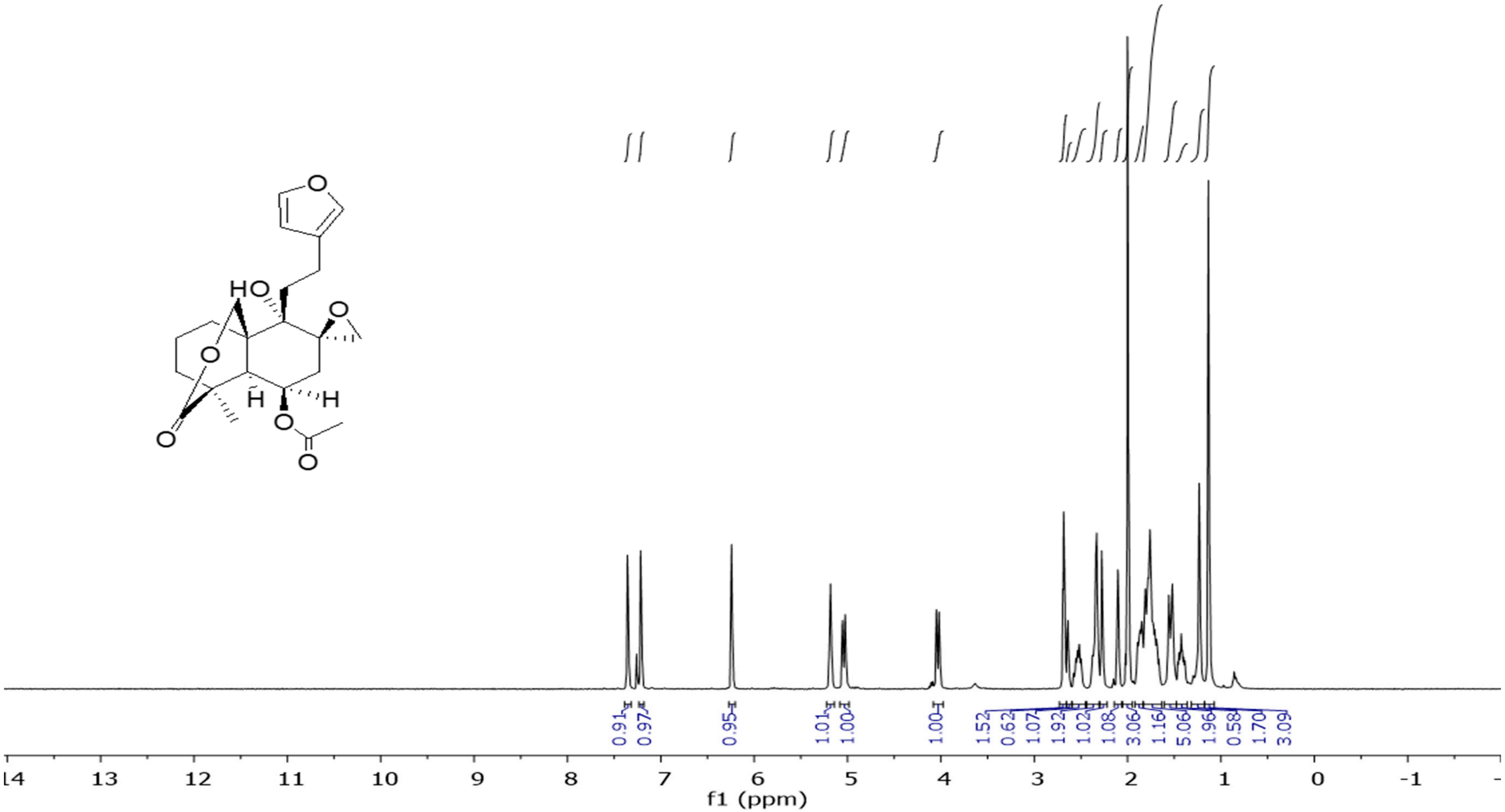

Spectrum D1: ^{1}H NMR spectrum of compound 4 (nepetaefuran)

Appendix 17: ¹³C NMR spectrum of nepetaefuran (4) in $CDCl_3$

	ppm	Intensity
1	176.30	15.4
2	170.55	17.1
3	143.56	38.8
4	138.81	38.3
5	124.24	19.2
6	110.66	36.8
7	74.51	27.5
8	73.63	29.7
9	68.06	32.4
10	56.75	28.4
11	46.96	27.5
12	46.45	33.1
13	42.08	27.8
14	41.19	29.3
15	39.96	27.7
16	32.52	28.2
17	32.34	28.9
18	25.88	28.8
19	22.47	30.7
20	21.08	35.3
21	20.48	55.0

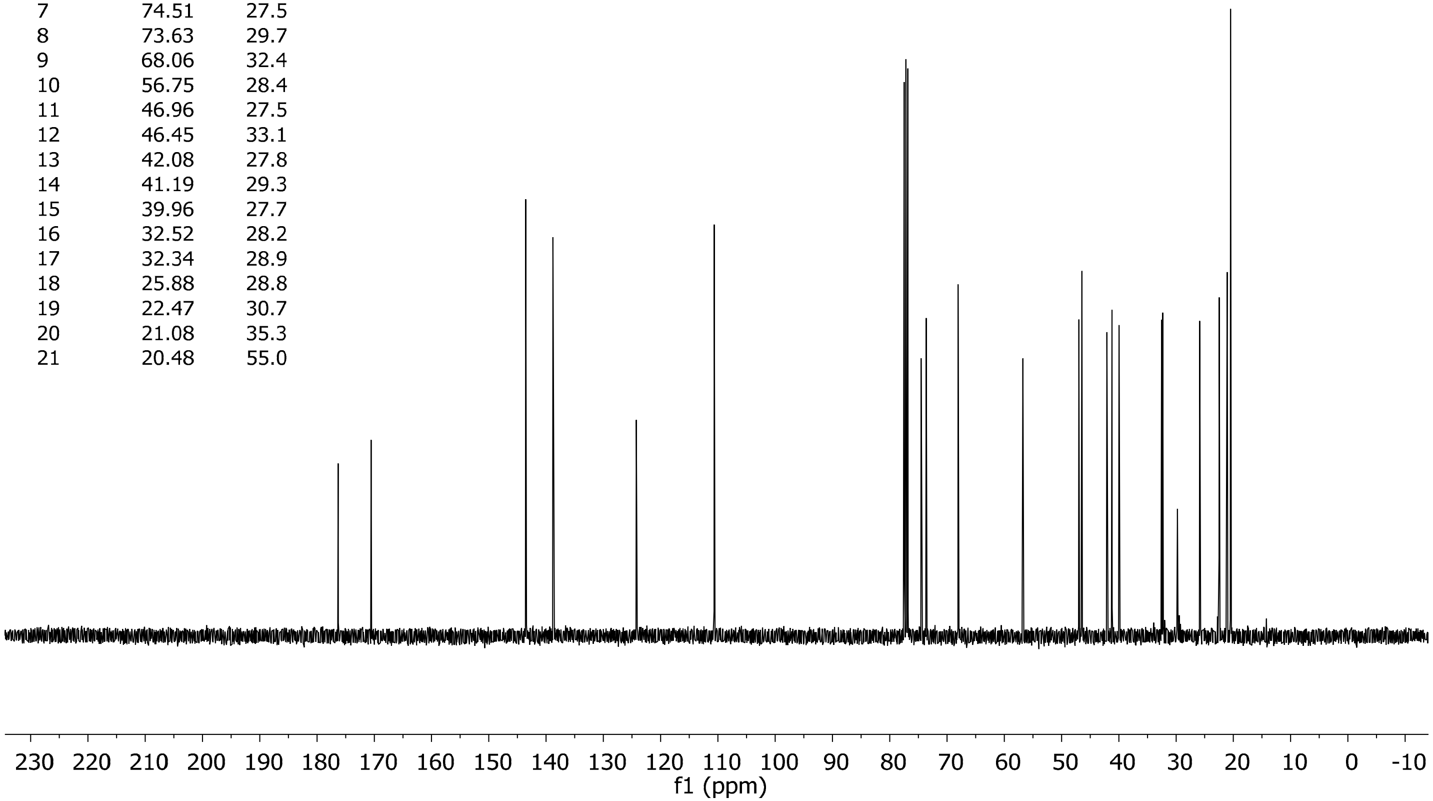

Spectrum D2: ¹³C NMR spectrum of compound 4 (nepetaefuran)

Appendix 18: IR spectrum of nepetaefuran (4)

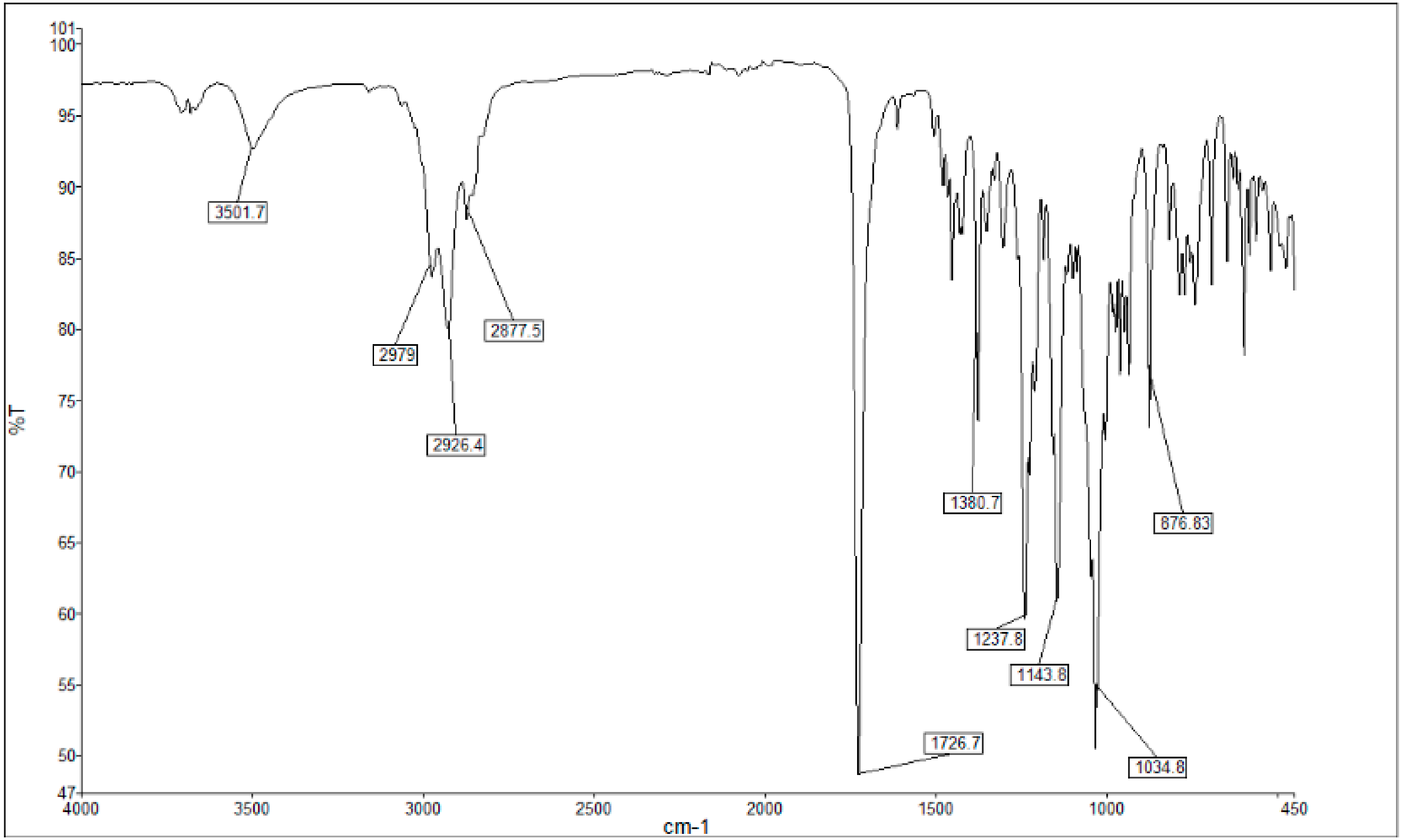

Spectrum D3: IR spectrum of compound 4 (nepetaefuran)

Appendix 19: MS spectrum of nepetaefuran (4)

Elemental Composition

File Edit View Process Help

Single Mass Analysis

Tolerance = 50.0 PPM / DBE: min = -1.5, max = 100.0

Element prediction: Off

Number of isotope peaks used for i-FIT = 3

Monoisotopic Mass, Even Electron Ions

241 formula(e) evaluated with 11 results within limits (up to 100 closest results for each mass)

Elements Used:

Mass	Calc. Mass	mDa	PPM	DBE	Formula	i-FIT	i-FIT Norm	Fit Conf %	C	H	N	O
405.1908	405.1913	-0.5	-1.2	8.5	C22 H29 O7	97.8	0.046	95.54	22	29		7
	405.1873	3.5	8.6	4.5	C17 H29 N2 O9	103.7	5.992	0.25	17	29	2	9
	405.1855	5.3	13.1	17.5	C29 H25 O2	107.2	9.469	0.01	29	25		2
	405.1967	-5.9	-14.6	17.5	C28 H25 N2 O	106.6	8.903	0.01	28	25	2	1
	405.1972	-6.4	-15.8	-0.5	C15 H33 O12	105.7	7.952	0.04	15	33		12
	405.1814	9.4	23.2	13.5	C24 H25 N2 O4	102.6	4.875	0.76	24	25	2	4
	405.2026	-11.8	-29.1	8.5	C21 H29 N2 O6	101.1	3.416	3.28	21	29	2	6
	405.1761	14.7	36.3	4.5	C18 H29 O10	105.2	7.501	0.06	18	29		10
	405.2066	-15.8	-39.0	12.5	C26 H29 O4	105.4	7.639	0.05	26	29		4

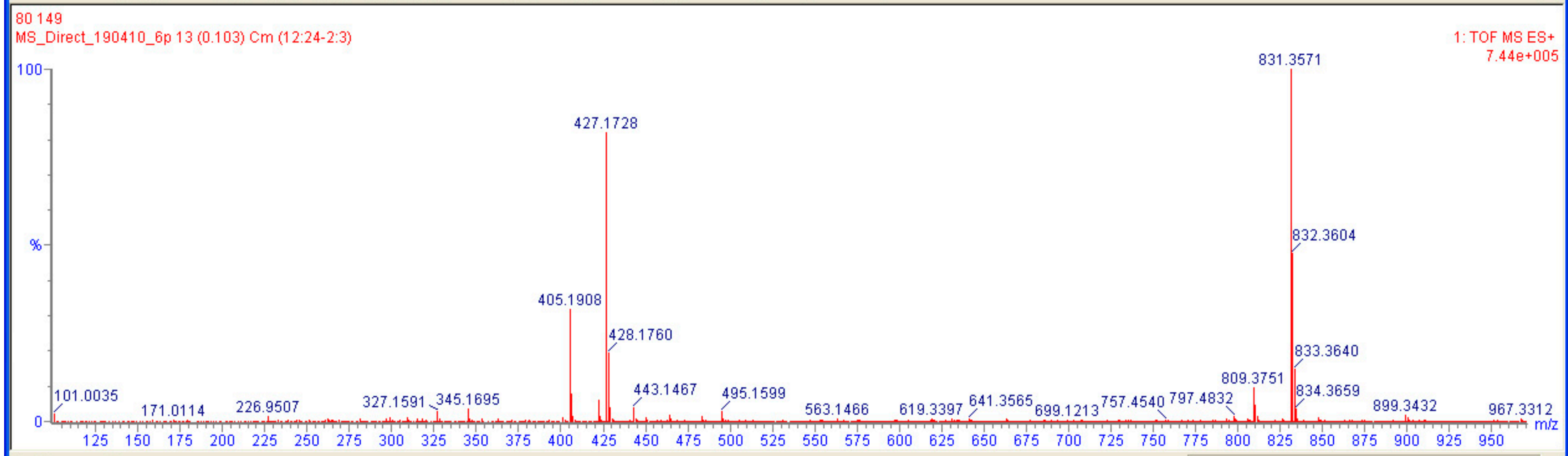

For Help, press F1

Spectrum D4: TOF-MS spectrum of compound 4 (nepetaefuran)

Appendix 20: ^1H NMR spectrum of leonotin (5) in $CDCl_3$

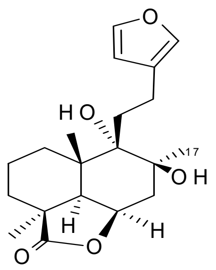

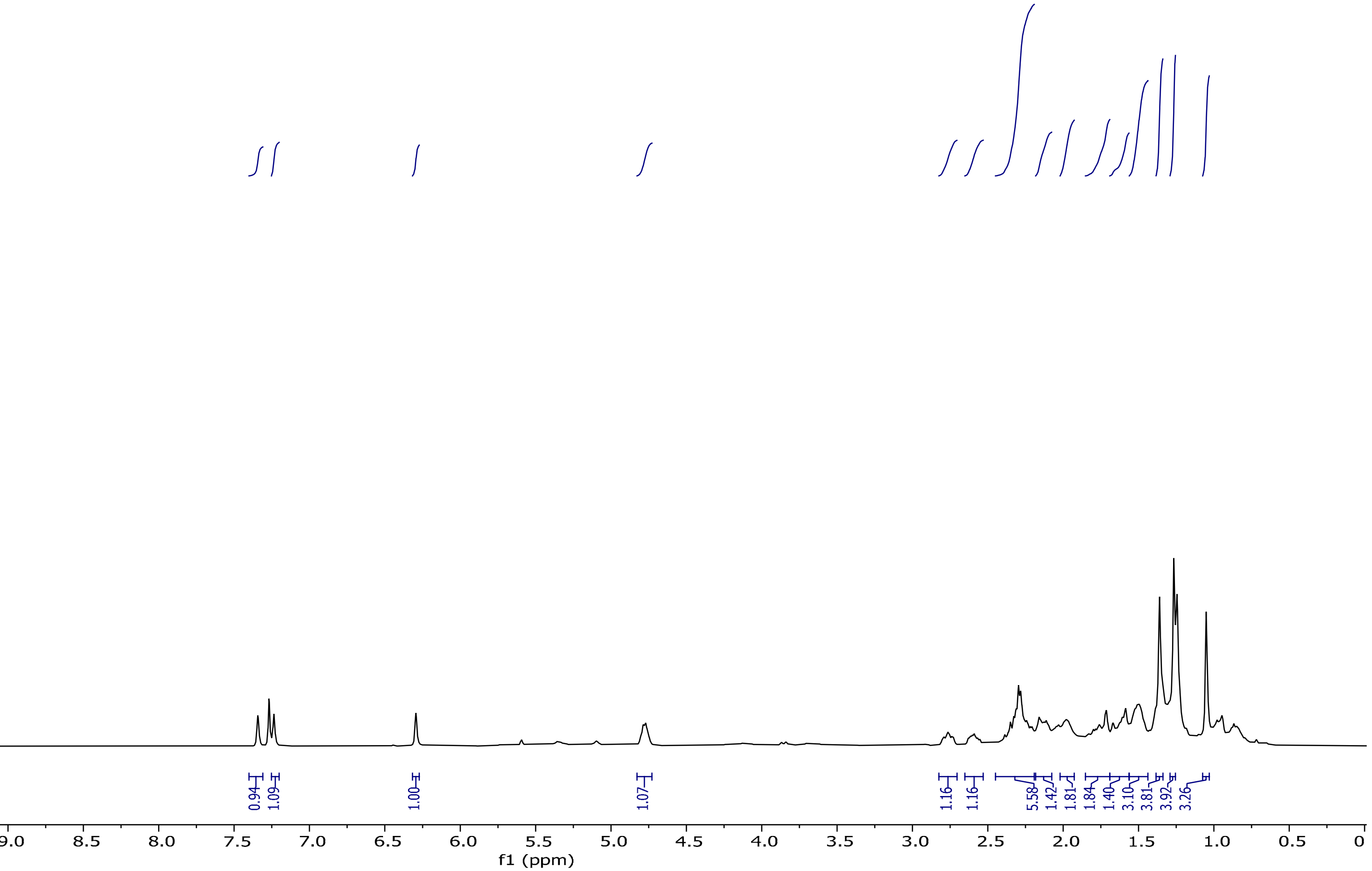

Spectrum E1: ^1H NMR spectrum of compound 5 (Leonotin)

Appendix 21: ^{13}C NMR spectrum of leonotin (5) in $CDCl_3$

	ppm	Intensity
1	182.85	2.9
2	142.97	15.1
3	138.60	15.7
4	125.47	5.9
5	110.91	11.3
6	79.64	10.2
7	75.47	11.0
8	74.70	8.2
9	43.86	14.8
10	42.34	9.3
11	42.01	12.5
12	40.81	10.1
13	32.10	12.5
14	31.33	13.6
15	30.79	14.9
16	28.72	13.5
17	25.42	15.7
18	21.00	15.9
19	20.69	13.9
20	17.84	15.5

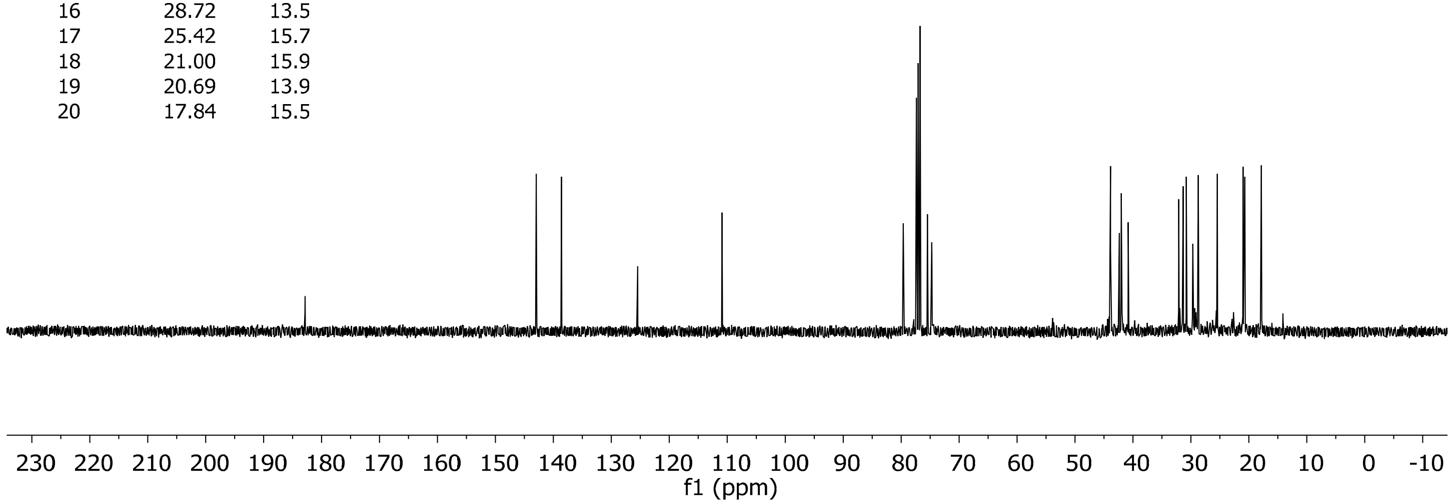

Spectrum E2: ^{13}C NMR spectrum of compound 5 (Leonotin)

Appendix 22: IR spectrum of leonotin (5)

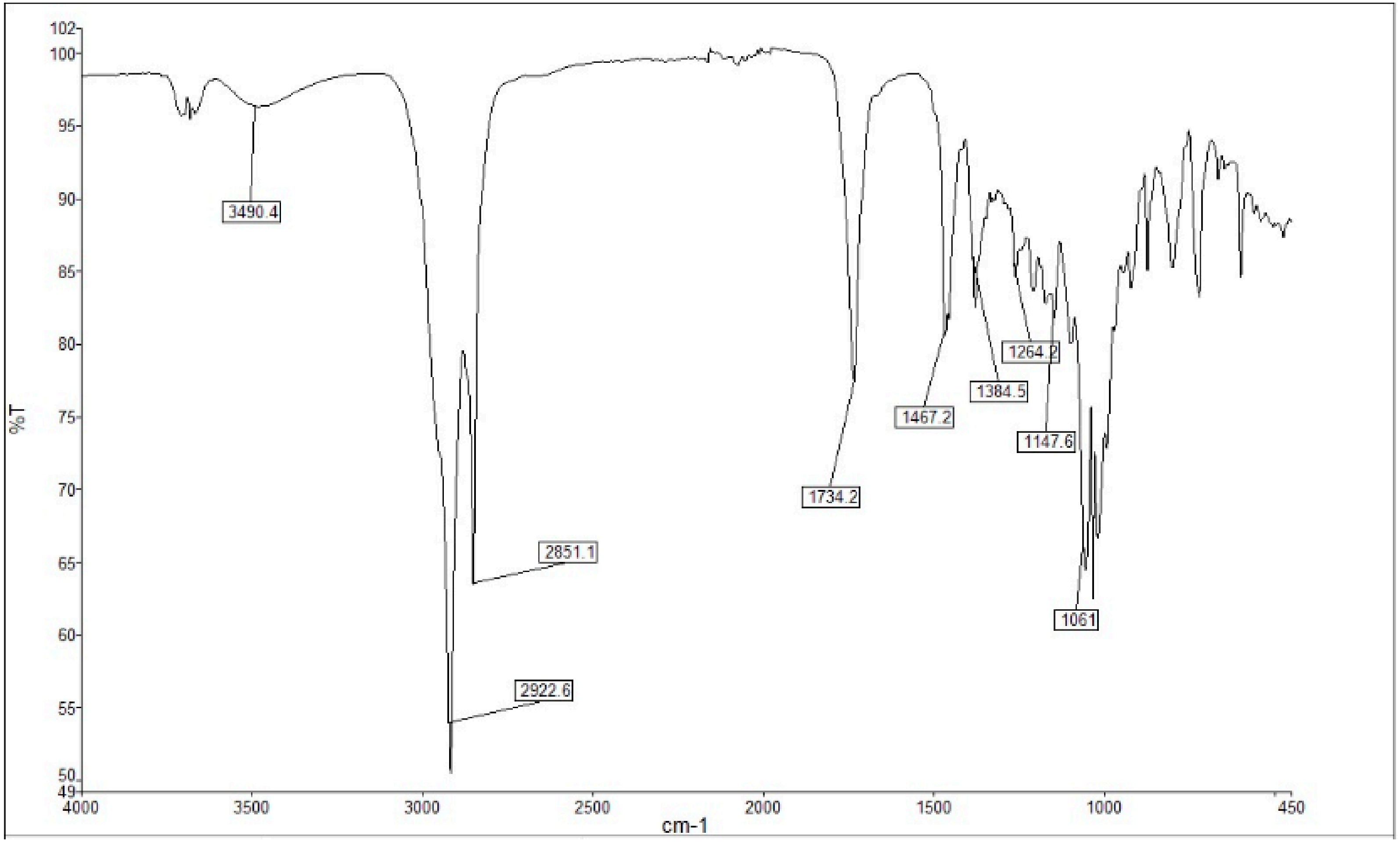

Spectrum E3: IR spectrum of compound 5 (Leonotin)

Appendix 23: MS spectrum of leonotin (5)

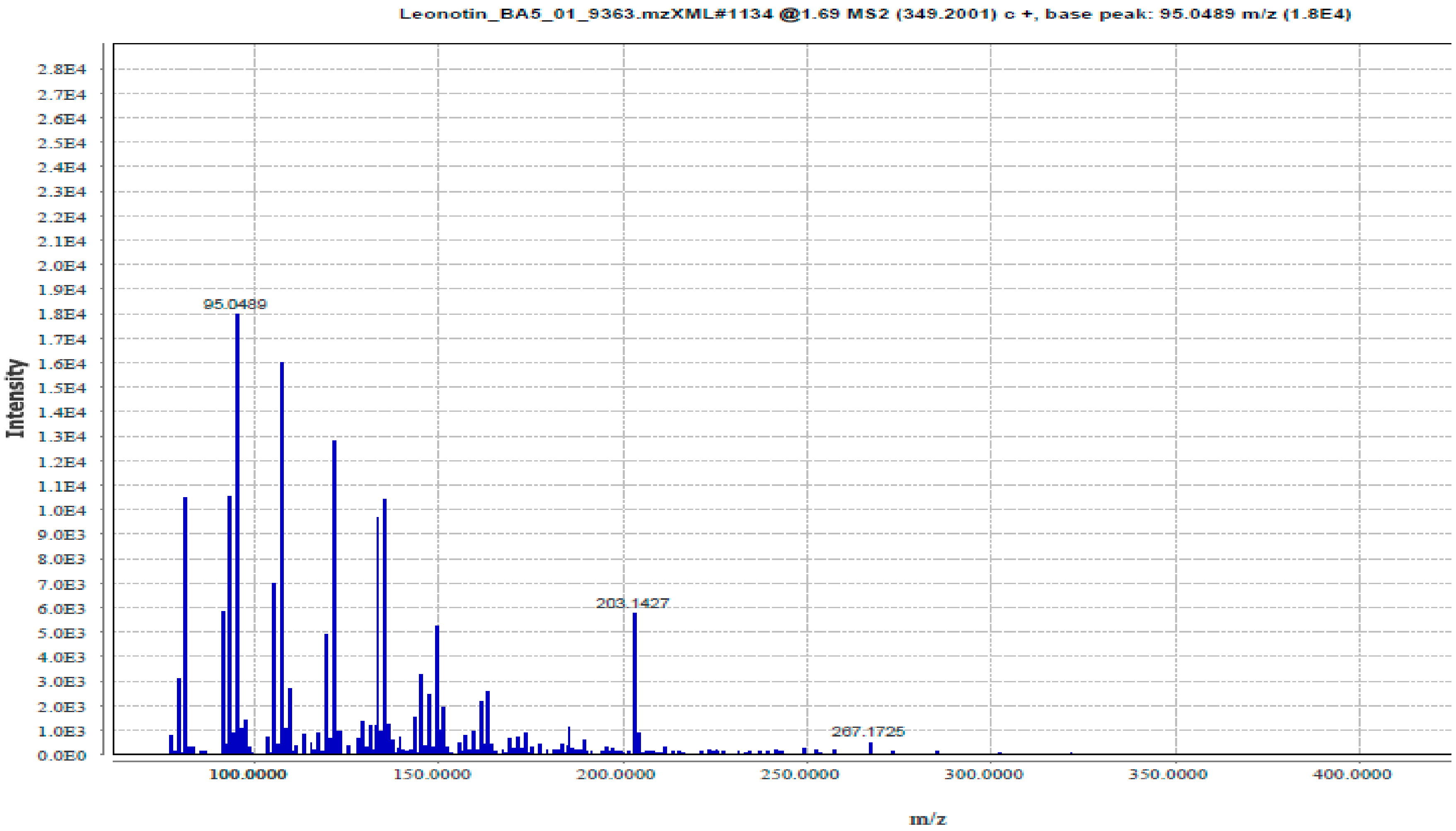

Spectrum E4: MS spectrum of compound 5 (Leonotin)

Appendix 24: ^1H NMR spectrum of leonotinin (6) in $CDCl_3$

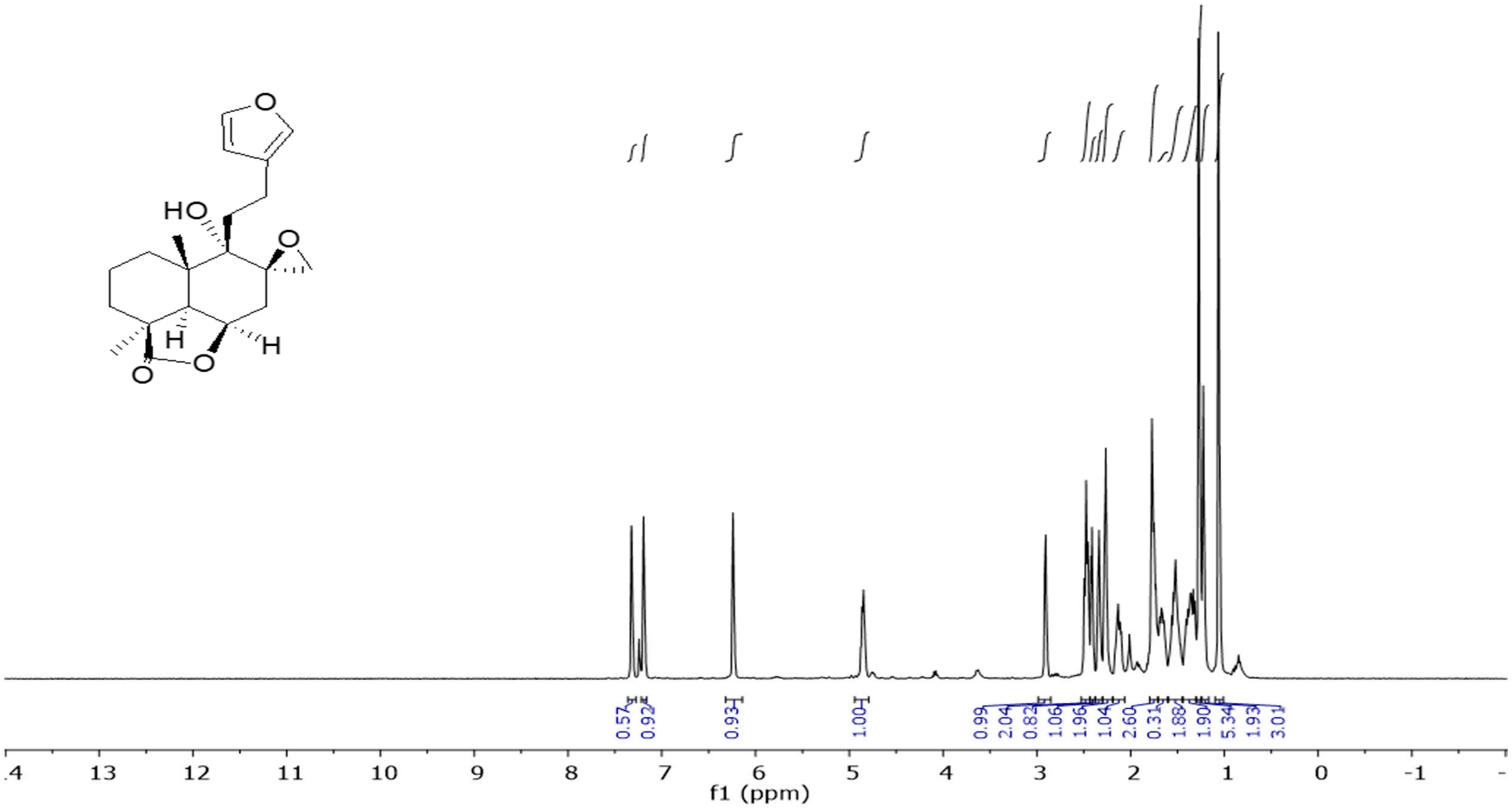

Spectrum F1: ^1H NMR spectrum of compound 6 (Leonotinin)

Appendix 25: ^{13}C NMR spectrum of leonotinin (6) in $CDCl_3$

	ppm	Intensity
1	182.60	5.1
2	143.14	17.7
3	138.65	16.2
4	124.91	6.3
5	110.73	15.4
6	76.10	8.4
7	74.39	16.5
8	59.31	8.8
9	50.46	16.8
10	45.11	18.1
11	42.82	10.3
12	40.65	9.4
13	32.96	17.5
14	29.91	18.7
15	29.70	19.0
16	28.65	18.2
17	24.38	18.2
18	20.82	17.6
19	20.23	17.9
20	18.08	18.9

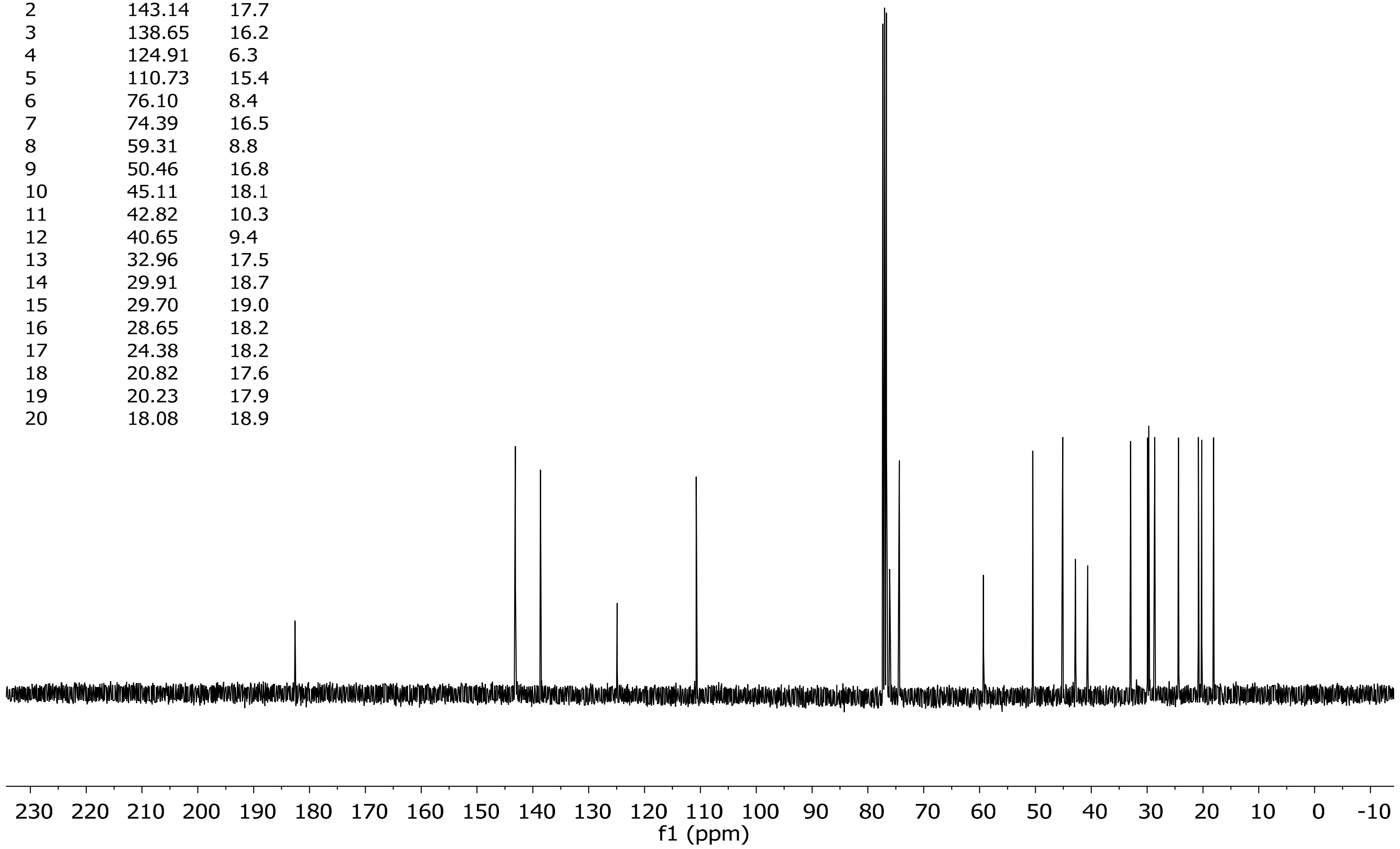

Spectrum F2: ^{13}C NMR spectrum of compound 6 (Leonotinin)

Appendix 26: IR spectrum of leonotinin (6)

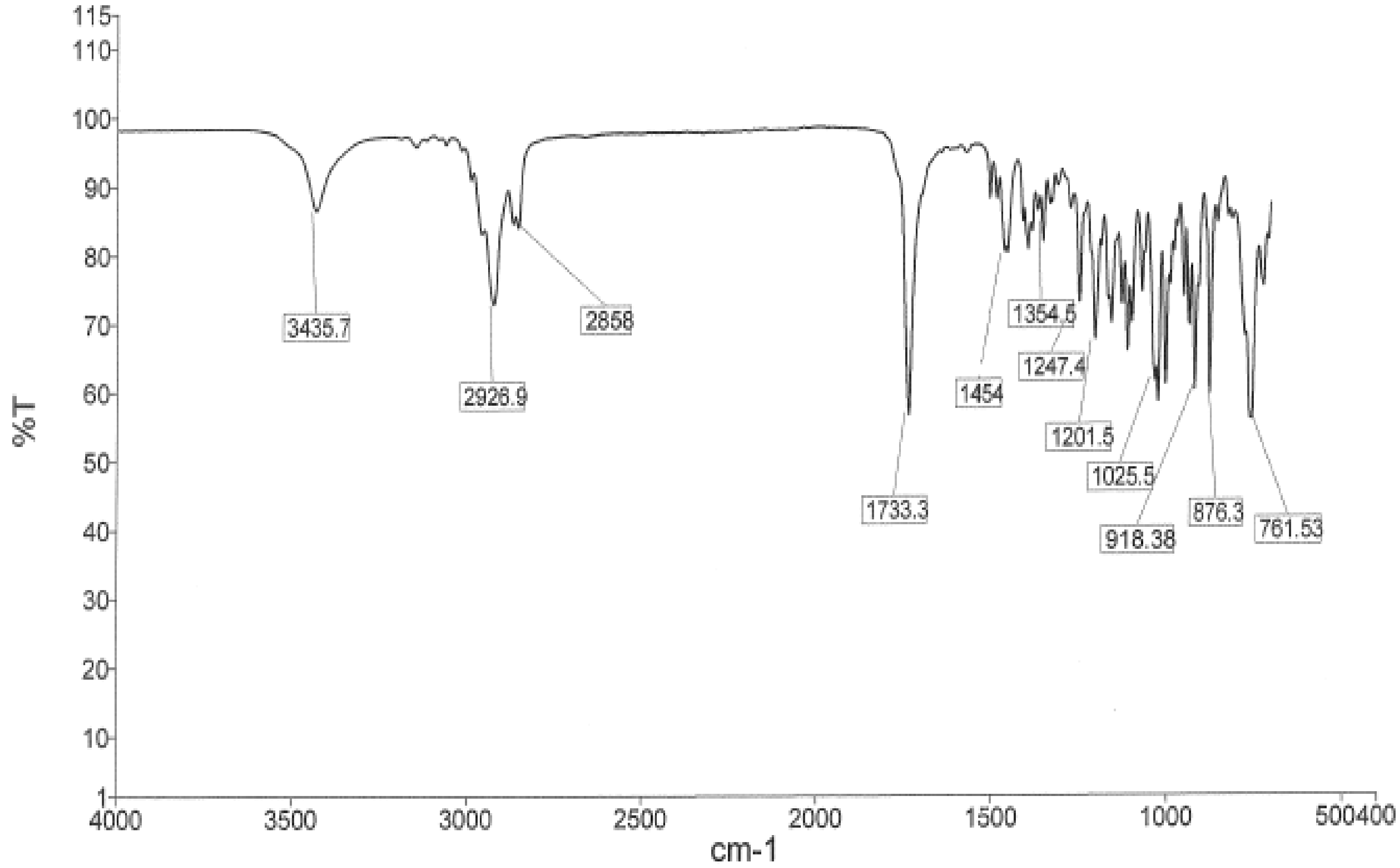

Spectrum F3: IR spectrum of compound 6 (Leonotinin)

Appendix 27: MS spectrum of leonotinin (6)

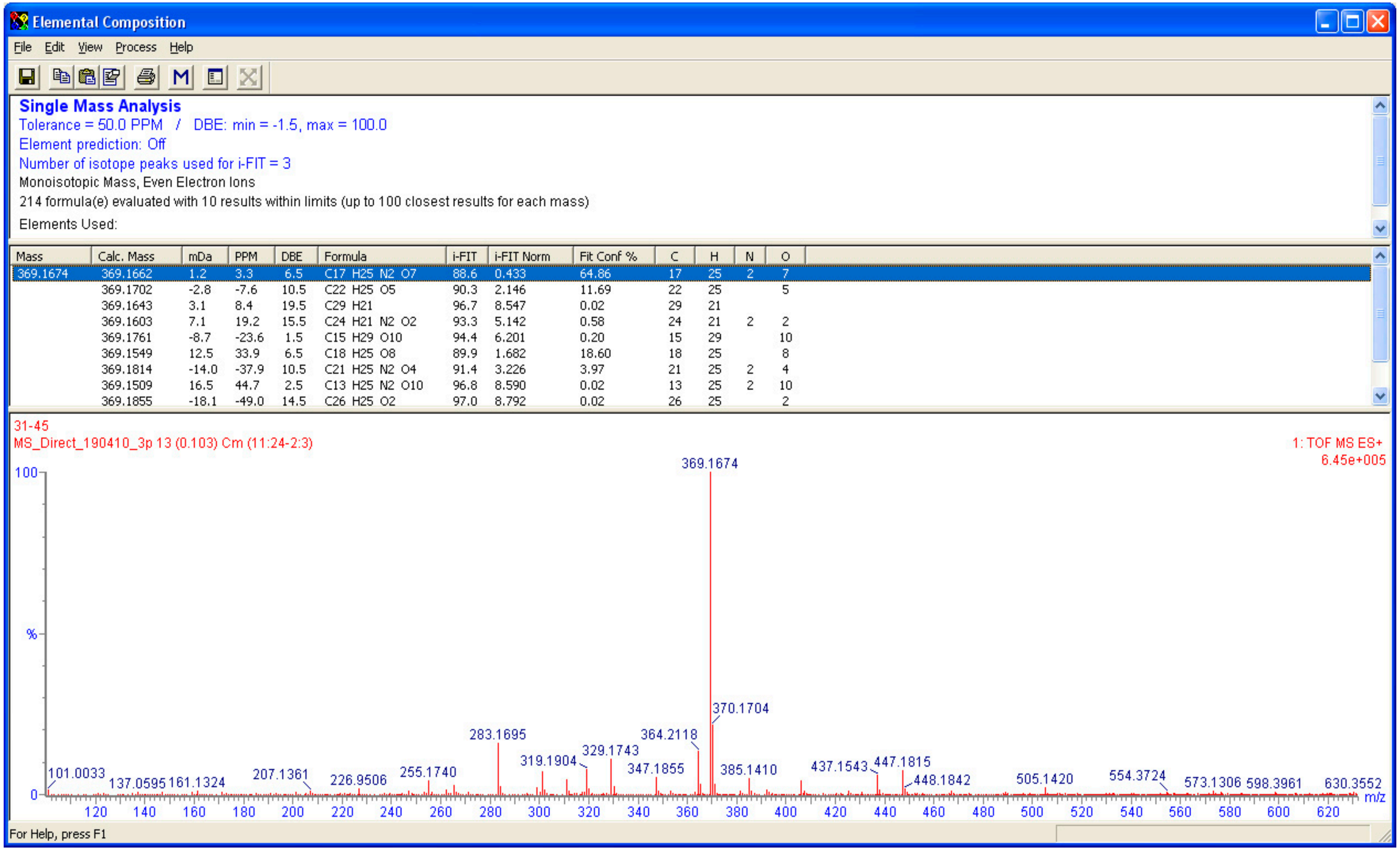

Mass	Calc. Mass	mDa	PPM	DBE	Formula	i-FIT	i-FIT Norm	Fit Conf %	C	H	N	O
369.1674	369.1662	1.2	3.3	6.5	C17 H25 N2 O7	88.6	0.433	64.86	17	25	2	7
	369.1702	-2.8	-7.6	10.5	C22 H25 O5	90.3	2.146	11.69	22	25		5
	369.1643	3.1	8.4	19.5	C29 H21	96.7	8.547	0.02	29	21		
	369.1603	7.1	19.2	15.5	C24 H21 N2 O2	93.3	5.142	0.58	24	21	2	2
	369.1761	-8.7	-23.6	1.5	C15 H29 O10	94.4	6.201	0.20	15	29		10
	369.1549	12.5	33.9	6.5	C18 H25 O8	89.9	1.682	18.60	18	25		8
	369.1814	-14.0	-37.9	10.5	C21 H25 N2 O4	91.4	3.226	3.97	21	25	2	4
	369.1509	16.5	44.7	2.5	C13 H25 N2 O10	96.8	8.590	0.02	13	25	2	10
	369.1855	-18.1	-49.0	14.5	C26 H25 O2	97.0	8.792	0.02	26	25		2

Spectrum F4: MS spectrum of compound 6 (Leonotinin)

Table S1 ^{13}C (δ_c) (100.6 MHz) NMR data for compounds **1-6** in $CDCl_3$.

NO.	1	2	3	4	5	6
**1**	33.07	33.29	32.86	32.52	31.44	29.85
**2**	20.32	20.35	20.39	20.48	17.95	18.22
**3**	39.69	39.61	39.71	39.96	28.83	28.79
**4**	40.77	40.74	41.08	42.08	42.45	42.96
**5**	46.98	46.91	46.76	46.45	43.97	45.25
**6**	67.97	67.92	68.36	68.06	74.81	74.53
**7**	32.43	32.47	33.32	32.34	42.12	33.10
**8**	56.44	56.07	30.16	56.75	75.58	59.45
**9**	85.92	85.95	74.87	74.51	79.75	76.24
**10**	41.02	41.03	40.96	41.19	40.92	40.79
**11**	23.63	23.54	35.04	25.88	32.21	30.05
**12**	37.46	37.51	20.49	20.36	20.80	20.37
**13**	92.41	92.73	124.42	124.24	125.58	125.05
**14**	107.01	105.50	110.51	110.66	111.01	110.87
**15**	149.43	149.45	143.21	143.56	143.08	143.29
**16**	80.07	81.11	138.54	138.81	138.71	138.79
**17**	47.53	47.16	15.60	46.96	30.90	50.60
**18**	22.21	22.21	22.37	22.47	25.53	24.52
**19**	175.91	175.85	175.95	176.29	182.96	182.75
**20**	73.90	73.80	75.88	73.63	21.10	20.96
**21**	170.33	170.34	170.21	170.55		
**22**	20.95	20.95	21.01	21.08		

Table S2: ^{1}H (δ_H, *mult*, (*J*, Hz) NMR (400 MHz) data for compounds **1-6** in $CDCl_3$.

	**1**	**2**	**3**	**4**	**5**	**6**
**1α**	1.80 (1H, m)	1.76 (1H, m)	1.88 (1H, m)	1.86 (1H, m)	1.50 (1H, m)	1.52 (1H, m)
**1B**	1.73 (1H, m)	1.72 (1H, m)	1.78 (1H, m)	1.74 (1H, m)	1.39 (1H, m)	1.33 (1H, m)
**2A**	1.80 (1H, m)	1.76 (1H, m)	1.77 (1H, m)	1.76 (2H, m)	1.63 (1H, m)	1.51 (1H, m)
**2B**	1.80 (1H, m)	1.75 (1H, m)	1.77 (1H, m)		1.51 (1H, m)	1.67 (1H, m)
**3A**	1.54 (1H, m)	1.50 (1H, m)	1.5 (1H, m)	1.50 (1H, m)	1.35 (1H, m)	1.37 (1H, m)
**3B**	1.84 (1H, m)	1.81 (1H, m)	1.83 (1H, m)	1.82 (1H, m)	2.12 (1H, m)	2.13 (1H, m)
**4**	-	-	-	-	-	-
**5**	2.01 (1H, d, *J* = 3.6 Hz)	2.00 (1H, d, *J* = 3.5 Hz)	2.06 (1H, m)	2.11 (1H, d, *J* = 3.3 Hz)	2.29 (1H, d, *J* = 6.1 Hz)	2.33 (1H, d, *J* = 4.9 Hz)
**6**	5.18 (1H, d, *J* = 3.4 Hz)	5.12 (1H, t, *J* = 3.3 Hz)	5.15 (1H, q, *J* = 3.3 Hz)	5.16 (1H, q, *J* = 3.2 Hz)	4.78 (1H, dt, *J* = 8.8, 6.2 Hz)	4.85 (1H, dt, *J* = 7.5, 5.0 Hz)
**7A**	2.63 (1H, dd, *J* = 15.4, 3.4 Hz)	2.65 (1H, (dd, *J* = 15.2, 3.4 Hz)	1.75 (1H, m)	2.64 (1H, d, *J* = 3.2 Hz)	2.34 (1H, m)	2.27 (2H, brd, *J* = 5.23 Hz)
**7B**	1.59 (1H, dd, *J* = 15.4, 3.4 Hz)	1.61 (1H, dd, *J* = 15.2, 3.4 Hz)	1.71 (1H, m)	1.52 (1H, dd, *J* = 15.6, 2.9 Hz)	2.34 (1H, m)	
**8**	-	-	2.12 (1H, dt, *J* = 12.1, 5.4 Hz)	-	-	-
**9**	-	-	-	-	-	-
**10**	-	-	-	-	-	-
**11A**	1.45 (1H, ddd, *J* = 14.0, 9.6, 4.9 Hz)	1.44 (1H, m)	1.89 (1H, m)	1.43 (1H, ddd, *J* = 14.9, 9.8, 4.9 Hz)	1.76 (1H, m)	1.75 (2H, brd, *J* = 8.3 Hz)
**11B**	1.76 (1H, m)	1.79 (1H, m)	1.74 (1H, m)	1.68 (1H, m)	2.25 (1H, m)	
**12A**	1.95 (1H, m)	1.94 (1H, m)	2.44 (1H, tt, *J* = 20.2, 10.0 Hz)	2.34 (1H, m)	2.59 (1H, ddd, *J* = 15.0, 10.5, 5.8 Hz)	2.47 (2H, t, *J* = 8.0 Hz)
**12B**	2.18 (1H, m)	2.16 (1H, m)	2.44 (1H, tt, *J* = 20.2, 10.0 Hz)	2.53 (1H, m)	2.77 (1H, ddd, *J* = 15.4, 10.9, 4.7 Hz)	
**13**	-	-	-	-	-	-
**14**	4.88 (1H, d, *J* = 2.7 Hz)	5.01 (1H, d, *J* = 2.5 Hz)	6.25 (1H, brs)	6.24 (1H, m)	6.3 (1H, brs)	6.24 (1H, brs)
**15**	6.48 (1H, d, *J* = 2.7 Hz)	6.52 (1H, d, *J* = 2.6 Hz)	7.35 (1H, t, *J* = 1.70 Hz)	7.36 (1H, t, *J* = 1.8 Hz)	7.34 (1H, t, *J* = 1.6 Hz)	7.32 (1H, d, *J*= 1.9 Hz)
**16A**	4.03 (1H, d, *J* = 10.6 Hz)	3.95 (1H, d, J = 10.4 Hz)	7.22 (1H, s)	7.22 (1H, brs)	7.24 (1H, brs)	7.19 (1H, brs)
**16B**	4.43 (1H, d, *J* = 10.6 Hz)	4.17 (1H, d, *J* = 10.41 Hz)				

**17A**	2.34 (1H, d, *J* = 3.9 Hz)	2.30 (1H, d, *J* = 3.9 Hz)	0.97 (3H, d, *J* = 5.7 Hz)	2.33 (1H, d, *J* = 3.8 Hz)	1.36 (3H, s)	2.41 (1H, d, *J* = 4.6 Hz)
**17B**	2.67 (1H, d, *J* = 3.9 Hz)	2.65 (1H, d, *J* = 3.9 Hz)		2.68 (1H, d, *J* = 3.8 Hz)		2.9 (1H, d, *J* = 4.7 Hz)
**18**	1.07 (3H, s)	1.08 (3H, s)	1.25 (3H, s)	1.11 (3H, s)	1.27 (3H, s)	1.27 (3H, s)
**19**	-	-	-	-	-	-
**20A**	3.97 (1H, d, *J* = 11.7 Hz)	3.93 (1H, d, *J* = 11.6 Hz)	4.26 (1H, d, *J* = 11.2 Hz)	4.03 (1H, d, *J* = 11.7 Hz)	1.05 (3H, s)	1.06 (3H, s)
**20B**	5.10 (1H, d, *J* = 11.7 Hz)	5.02 (1H, d, *J* = 11.6 Hz)	4.68 (1H, d, *J* = 11.2 Hz)	5.01 (1H, d, *J* = 11.8 Hz)		
**21**	-	-	-	-		
**22**	1.98 (3H, s)	1.96 (3H, s)	2.02 (3H, s)	1.97 (3H, s)		
